# Identification of genetic mechanisms underlying lipid metabolism-mediated tumor immunity in head and neck squamous cell carcinoma

**DOI:** 10.1186/s12920-023-01543-6

**Published:** 2023-05-20

**Authors:** Shaokun Liu, Shuning Wang, Zhenlin Wang

**Affiliations:** 1 Department of Otorhinolaryngology Head and Neck Surgery, Xuanwu Hospital Capital Medical University, No. 45 Changchun Street, Xicheng District, Beijing, 100053 China; 2 Capital Medical University, No.10 Xitou Tiao, You’an Menwai, Fengtai District, Beijing, 10069 China

**Keywords:** Lipid metabolism, Tumor immunity, Head and neck squamous carcinoma, Bioinformatics

## Abstract

**Objective:**

To identify the genetic mechanisms underlying lipid metabolism-mediated tumor immunity in head and neck squamous carcinoma (HNSC).

**Materials and methods:**

RNA sequencing data and clinical characteristics of HNSC patients were procured from The Cancer Genome Atlas (TCGA) database. Lipid metabolism-related genes were collected from KEGG and MSigDB databases. Immune cells and immune-related genes were obtained from the TISIDB database. The differentially expressed genes (DEGs) in HNSC were identified and weighted correlation network analysis (WGCNA) was performed to identify the significant gene modules. Lasso regression analysis was performed to identify hub genes. The differential gene expression pattern, diagnostic values, relationships with clinical features, prognostic values, relationships with tumor mutation burden (TMB), and signaling pathways involved, were each investigated.

**Results:**

One thousand six hundred sixty-eight DEGs were identified as dysregulated between HNSC tumor samples and healthy control head and neck samples. WGCNA analysis and Lasso regression analysis identified 8 hub genes, including 3 immune-related genes (PLA2G2D, TNFAIP8L2 and CYP27A1) and 5 lipid metabolism-related genes (FOXP3, IL21R, ITGAL, TRAF1 and WIPF1). Except CYP27A1, the other hub genes were upregulated in HNSC as compared with healthy control samples, and a low expression of these hub genes indicated a higher risk of death in HNSC. Except PLA2G2D, all other hub genes were significantly and negatively related with TMB in HNSC. The hub genes were implicated in several immune-related signaling pathways including T cell receptor signaling, Th17 cell differentiation, and natural killer (NK) cell mediated cytotoxicity.

**Conclusion:**

Three immune genes (PLA2G2D, TNFAIP8L2, and CYP27A1) and immune-related pathways (T cell receptor signaling, Th17 cell differentiation, and natural killer (NK) cell mediated cytotoxicity) were predicted to play significant roles in the lipid metabolism-mediated tumor immunity in HNSC.

## Introduction

Imbalance in lipid metabolism homeostasis is an important metabolic hallmark of cancer [1, 2]. Water-insoluble molecules, e.g., triacylglycerides, phosphoglycerides, sterols and sphingolipids, formed by lipids are principal components of biological membranes, and critical building blocks used for energy storage. In cancer cells, lipid metabolism and related processes are critical for energy generation along with the maintenance of membrane components and signaling molecules necessary for various cancer cell processes such as cell proliferation, survival, invasion, metastasis [1, 2]. The impairment of lipid metabolic homeostasis not only influences the cellular processes of cancer cells, but also play critical roles in regulating anti-tumor immunity [3]. By secreting lipid-derived signaling molecules in the tumor microenvironment (TME), tumor cells can alter the metabolic programming of immune cells toward fatty acid oxidation, which is associated with pro-tumorigenic and immune-suppressive phenotypes of immune cells [4].

Head and neck squamous cell carcinoma (HNSCC) accounts for more than 90% of all head and neck cancers worldwide, ranking sixth in the world [5]. A recent reported that 23 genes associated with lipid metabolism showed significant prognostic values in HNSCC, where 11 genes (ARSI, CYP27B1, CYP2D6, DGKG, DHCR7, LPIN1, PHYH, PIP5K1B, PLA2G2D, RDH16, and TRIB3) were associated with clinicopathological features (stage, gender, and pathological stage) [6]. However, this study [6] did not investigate the underlying genetic and immunological mechanisms involved in lipid metabolism-mediated regulation of tumor immunity in HNSC. Recent work has demonstrated that HNSC tumor cell-derived fatty acids can mediate the maturation of macrophages into a M2 phenotype which plays anti-inflammatory and pro-tumor role [7]. Another recent study found that lipid nanoparticle-based targeted delivery led to a decrease in the adenosine A2A receptor, which in turn increased chemotaxis and T cell infiltration [8]. Considering the emerging evidence regarding a pivotal role of lipid metabolism in regulating tumor immunity in HNSC, an improved understanding of the molecular mechanisms involved could provide basis for novel therapeutic strategies to overcome tumor-induced immunosuppression in HNSC.

The advances in computational biology approaches have resulted in several analytical approaches that could generate insights into molecular processes involved in lipid metabolism-mediated regulation of tumor immunity in HNSC. In the present study, we leveraged weighted gene co-expression network analysis (WGCNA), Lasso regression analysis, survival analysis, nomogram plot analysis, tumor mutation burden (TMB) analysis, and gene-pathway interaction analysis for this purpose. By comprehensively applying these analyses, the current study aimed to investigate the most significant mechanisms involved in lipid metabolism-mediated tumor immunity in HNSC.

## Materials and methods

### Procurement of HNSC datasets

We downloaded RNA-seq data of HNSC samples from TCGA-GDC (https://portal.gdc.cancer.gov/) [9], and the extracted gene expression values as a TPM (Transcripts Per Million) dataset. We selected the samples with sample numbers beginning with 01 and 11 for analysis, which was based on the sample type codes in the TCGA code table (URL: https://gdc.cancer.gov/resources-tcga-users/tcga-code-tables/sample-type-codes). The samples with sample numbers starting with 01 comprised the Tumor group (such as TCGA-BA-5151-01A), and those with sample numbers starting with 11 comprised the Normal group (such as TCGA-CV-7406-11A). We also downloaded the relevant clinical information data and the SNV (simple nucleotide variation) dataset [10], where the data type of the SNV dataset was ‘Masked Somatic Mutation’.

### Procurement of lipid metabolism-associated genes and immune-related gene set

The lipid metabolism-related feature gene set was gathered from KEGG (Kyoto Encyclopedia of Genes and Genomes, http://www.kegg.jp/blastkoala/) [11] and MSigDB (Molecular Signatures Database v7.5.1, https://www.gsea-msigdb.org/gsea/msigdb/index.jsp) [12] using the following keywords; fatty acyls, glycerolipids, glycerophospholipids, sphingolipids, sterol lipids, prenol lipids, saccharolipids, and polyketides. We thus obtained 21 pathways related to lipid metabolism from the KEGG database, and 6 gene sets related to lipid metabolism from MSigDB database. After integrating the data from the two databases, a total of 1079 lipid metabolism-related genes and 27 pathways were finally obtained. We downloaded immune cells and related genes from the TISDB database (http://cis.hku.hk/TISIDB/download.php) [13], and obtained 28 immune cells and 782 immune-related genes.

### Data preprocessing

We selected tumor samples that contained clinical information, and then used the selected tumor samples and normal samples to form an expression matrix for subsequent analysis. We de-duplicated the duplicate genes in the expression matrix according to the mean, deleted the genes whose expression values were 0 in more than 50% of the samples, and performed log2 scaling on the gene expression values.

### Gene set variation analysis

Gene set variation analysis (GSVA) is a non-parametric and unsupervised method for identifying patterns in gene expression profiles that correlate with biological pathways [14]. We first downloaded datasets of pathways and genes from the MSigDB database [12]. We then merged the lipid metabolism-related genes and immune-related genes, extracted their expression values in HNSC samples. And performed GSVA enrichment analysis on the gene expression matrix to obtain the enrichment scores of pathways in each sample. Next, we applied the “limma” package [15] in R to perform differential expression analysis [16] for Tumor vs Normal sample groups. The thresholds used included |logFC|> 0.25 and *P*.adjust < 0.05, where log2FC > 0.25 indicated differentially up-regulated pathways, and log2FC < 0.25 indicated differentially down-regulated pathways.

### Univariate analysis to screen survival-related genes

We merged lipid metabolism-related genes and immune-related genes, and then extracted the gene expression values for the combined gene set in the Tumor group. The ‘survival’ package [17] in R was used to build a Cox proportional hazards regression model (Cox-PH) [18] for each gene. The genes identified by the Cox-PH univariate analysis with *P*-value < 0.05 were regarded as survival-related genes and included in the subsequent analysis.

### WGCNA for the significant module

The expression values of the survival-related genes in the tumor case group were obtained. The WGCNA package [19] in R was applied to establish a scale-free weight network for these genes and samples, and the consistency module was extracted. An appropriate adjacency matrix weight parameter β value was selected by setting the β value from 1 to 30, and then calculating the corresponding correlation coefficient and gene adjacency function mean of the dataset. The higher the correlation coefficient (R^2^) (maximum = 1), the closer the network is to a no network scale distribution. At the same time it is also necessary to ensure a certain degree of gene connectivity.

Based on the correlation coefficient and gene connectivity, we selected the β value, and then applied the TOMsimilarity method [20] to establish a given adjacency matrix based on the expression matrix. Next, we used the ‘dynamic cut tree’ algorithm to cut the given adjacency matrix and perform module mining and thereafter used the ‘mergeCloseModules’ method to merge modules with correlation coefficients greater than 0.8. We then obtained the *P* value of the genes in the univariate analysis and looked at the significance of each module. Among the modules obtained by WGCNA, the grey module comprised an unassigned genes module and was not included in the subsequent analysis. We counted the number of genes in other significant modules, and selected modules with both lipid metabolism-related genes and immune-related genes for the subsequent analysis.

### Hub gene screening in significant modules

Through WGCNA analysis, we obtained significant modules related to lipid metabolism genes and immune genes. We then extracted the expression values of these genes in the module in tumor and normal samples and performed differential expression analysis using limma in R [15] with the comparison method as Tumor vs Normal. The resultant genes with |logFC|> 0.5 and *P*.adjust < 0.01 were considered as the significant differently expressed genes (DEGs). Next, we used LASSO (Least absolute shrinkage and selection operator) logistic regression analysis [21] to further screen the DEGs. Pearson’s correlation coefficient analysis was applied to investigate the correlation between the lipid metabolism-related genes and immune-related genes. If a lipid metabolism-related gene was highly correlated with all immune-related genes, then we extracted this gene and recorded it as a ‘Hub’ gene. We extracted the expression levels of the Hub genes in tumor and normal samples and applied the Wilcoxon test to the Hub genes in different groups. Next, we applied ROC analysis [22] to test the prognostic values of the hub genes.

### DNA methylation analysis of immune-related hub genes

DNA methylation is currently the most studied epigenetic modification and is essential for promoting important biological processes such as embryonic development, genomic imprinting and X chromosome inactivation. MethSurv (URL: https://biit.cs.ut.ee/methsurv/) is an intuitive web-based tool for multivariable survival analysis based on CpG methylation patterns [23]. The MethSurv tool was used to analyze methylation level differences in the immune-related hub genes between groups of samples with different clinical characteristics. Using the common regions of the immune-related hub genes, the relationship between these genes’ methylation level and sample clinical characteristics was analyzed. In this analysis, DNA methylation values were represented using beta values (ranging from 0 to 1) [24]. The parameters used in the methylation analysis are listed in Table 1. In addition, MethSurv was also used to analyze the influence of immune-related hub genes’ DNA methylation on the survival prognosis of TCGA-HNSC [25].

**Table 1:** The parameters used in the methylation analysis

Gene	Cancer	Relation to island	Genomic region	CpG site	Split by
PLA2G2D	HNSC TCGA March 2017	Open_Sea	Body	Cg14321743	Median
TNFAIP8L2	HNSC TCGA March 2017	Open_Sea	Body	Cg11825431	Median
CYP27A1	HNSC TCGA March 2017	Open_Sea	Body	Cg12806497	Median

### Immune cell infiltration analysis for immune-related hub genes

The expression values of the immune-related hub genes in HNSC tumor samples were collected and immune infiltration analysis was performed using Tumor Immune Estimation Resource (TIMER, URL: http://timer.cistrome.org/) database [26]. TIMER analyzes the enrichment scores of immune cells in different samples using 6 computational methods (e.g., CIBERSORT, CIBERSORT-ABS, EPIC, MCPCOUNTER, QUANTISEQ, and TIMER) [26–28]. The scores of the immune cells using the different methods were obtained, and then their relationship with immune cells was analyzed using Pearson’s correlation coefficient analysis [29]. An absolute r value between 0 and 0.3 indicated a weak linear relationship, that between 0.3 and 0.7 indicated a moderate relationship, and that between 0.7 and 1.0 indicated a strong relationship [29].

### Prediction of targeted small molecule drugs

The significant module associated with survival identified by the WGCNA analysis was used and a connectivity Map (CMap, URL: https://clue.io/) [30] was used to discover small molecule targets and functionally annotate genetic variants of disease genes [31]. Both upregulated genes (n ≥ 10) and downregulated genes (n ≥ 10) were uploaded to the CMap database to predict potential small molecular drug targets for the genes in the significant module. The top 10 drugs/molecules with positive normalized WTCS (weighted connectivity score) value and the top 10 drugs/molecules with negative normalized WTCS value were obtained using CMap [32].

### Multivariate analysis of hub genes

In order to investigate the relationship between the hub genes and survival outcomes of HNSC, we extracted the expression values of hub genes in tumor samples, and a Cox-PH model was built to predict OS and OS_Event using multivariate analysis. Thus, the risk scores for the hub genes were obtained. The samples were divided into high-risk and low-risk groups on based on the median risk scores and we examined whether low-risk survival differed at different time periods (3 years, 5 years, and 10 years). Next, we extracted the expression values of the hub genes in different risk groups and performed Wilcoxon’s test for different risk groups.

To analyze the relationship between clinical characteristics and high- and low- risk groups, the clinical characteristics and sample risk scores were integrated. We then constructed a nomogram plot by utilizing the nomogram method in the “rms” package [33] in R. The Wilcoxon’s test was applied to analyze whether there were significant differences in risk scores between different groups of clinical characteristics.

### Somatic mutation analysis of hub gene

Based on SNV data, we analyzed the mutation information regarding hub genes in the tumor samples and calculated the TMB score [34] of the tumor samples. We extracted the expression values of hub genes in HNSC samples and used the TMB scores for Pearson correlation coefficient analysis [29] to analyze the relationship between hub genes and TMB.

### Pathway analysis of hub genes

We obtained the genes related to all pathways from the KEGG database [11], and then extracted the pathways where the hub genes were represented. Next, we extracted all the genes under these pathways, and selected survival-related lipid metabolism genes and immune genes. Thereafter, we used Cytoscape (version 3.9.1) software [35] to construct a hub genes-pathways-survival genes interaction network.

## Results

### HNSC dataset

We obtained the HNSC dataset from TCGA and obtained 501 tumor samples and 44 normal samples. Table 2 summarizes the clinical features of the 501 HNSC tumor samples.

**Table 2:** Statistical summarize of clinical characteristics of HNSC

Characteristic	Category	Sample size
Overall survival(OS)	Within 3 years	368
	Within 5 years	449
	Within 10 years	490
	All	501
OS_event	Alive	283
	Dead	218
Gender	Female	133
	Male	368
Age	Old (Age > = 60)	279
	Young (Age < 60)	222
Clinical stages	Stage I	19
	Stage II	95
	Stage III	102
	Stage IV	271
	Total (Stage I–Stage IV)	487
Stages_T	T1	33
	T2	144
	T3	130
	T4	179
	Total (T1–T4)	486
Stages_M	M0	471
	M1	5
	Total (M0–M1)	476
Stages_N	N0	329
	N1	80
	N2	153
	N3	7
	Total (N0–N3)	479

### Gene set variation analysis

**Fig. 1:**
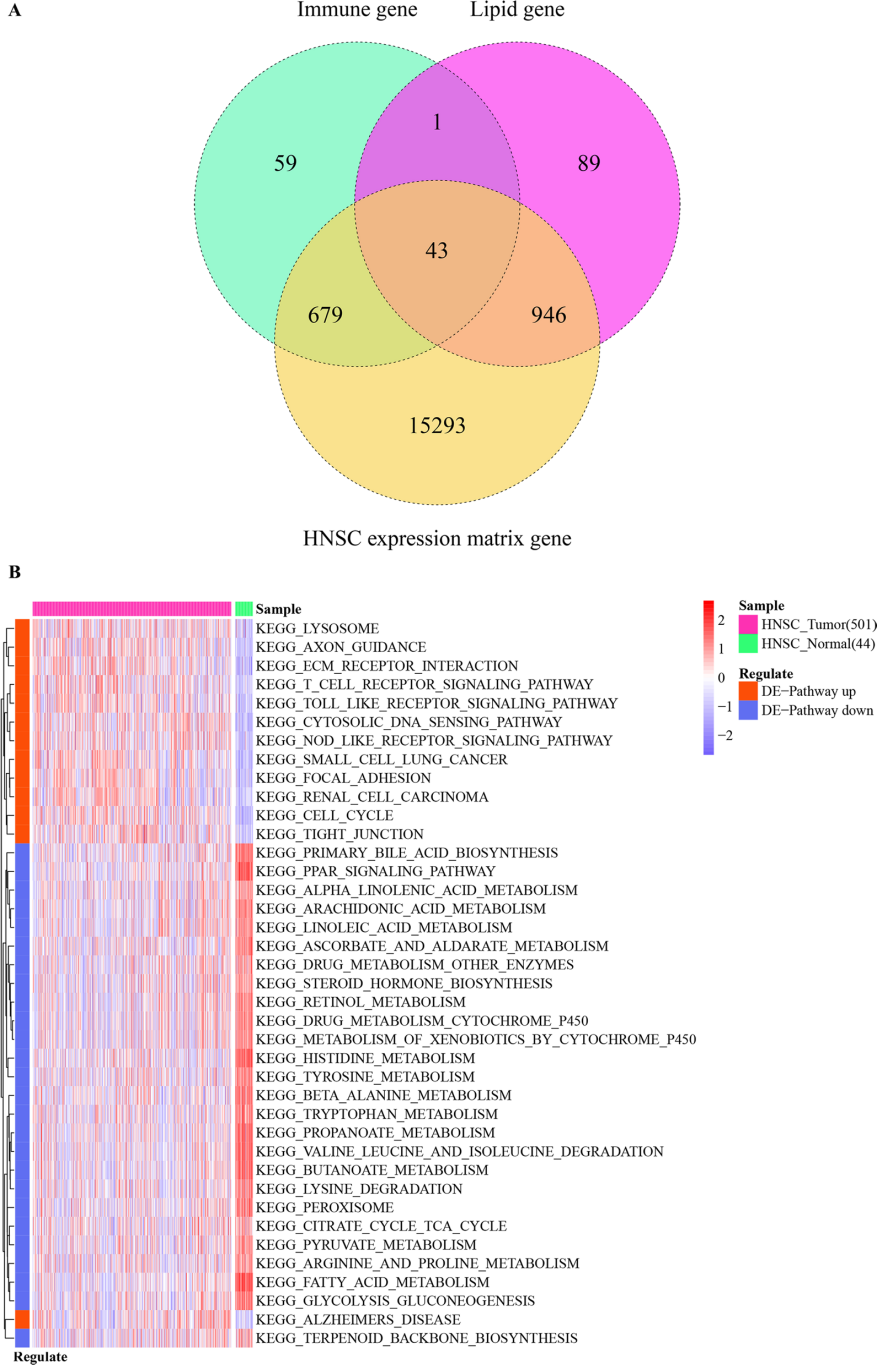
Functional analysis of the regulation of lipid metabolism-related genes and immune-related genes. **A** A Venn diagram depicting the overlap between lipid metabolism-related genes, immune-related genes, and DEGs dysregulated in HNSC. The circles with different colors represent the different groups of genes. 43 genes were found overlapping between lipid metabolism-related genes, immune-related genes, and HNSC-DEGs. **B** The functions of lipid metabolism- and immune-related genes revealed by functional enrichment analysis. Rose represents HNSC tumor samples, and fluorescent green represents healthy control samples; red represents up-regulated pathways and blue-purple represents down-regulated pathways. A darker color represents the greater significance

Fourty three common genes (Fig. 1A) were found overlapped between 1079 lipid metabolism-related genes, 782 immune-related genes, and 1688 HNSC-DEGs. These 1688 HNSC-DEGs regulated the T cell receptor signaling pathway, cytosolic dna sensing pathway, ecm receptor interaction, nod like receptor signaling pathway, toll like receptor signaling pathway, and metabolism of xenobiotics by cytochrome p450 among others (Fig. 1B).

### Screening of functional genes associated with survival by univariate analysis

**Fig. 2:**
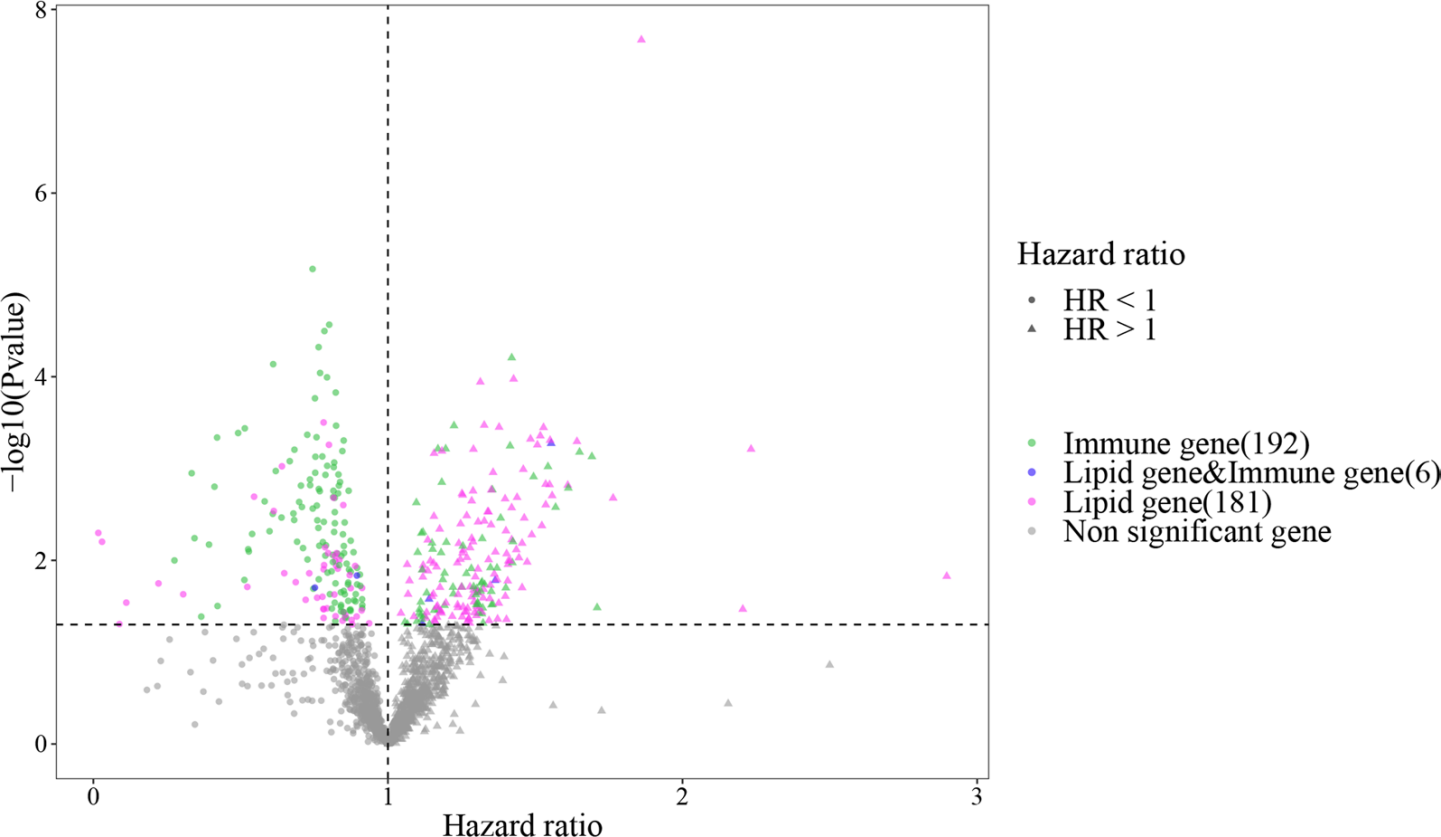
Volcano plot depicting the differential expression of survival-related genes. The x-axis represents the hazard ratio (HR), y-axis represents the negative log of the p value (usually base 10). The points with different colors represent different categories of genes including immune genes, lipid metabolism and immune-related genes, lipid metabolism-related genes, and non-significant genes. Circular points represent HR < 1 for the gene, while triangular points represent HR > 1 for the gene

Three hundred seventy-nine genes related to survival, including 192 immune-related genes and 181 lipid metabolism-related genes and 6 common genes (Fig. 2).

### Mining modules highly related to lipid metabolism- and immune-related genes

**Fig. 3:**
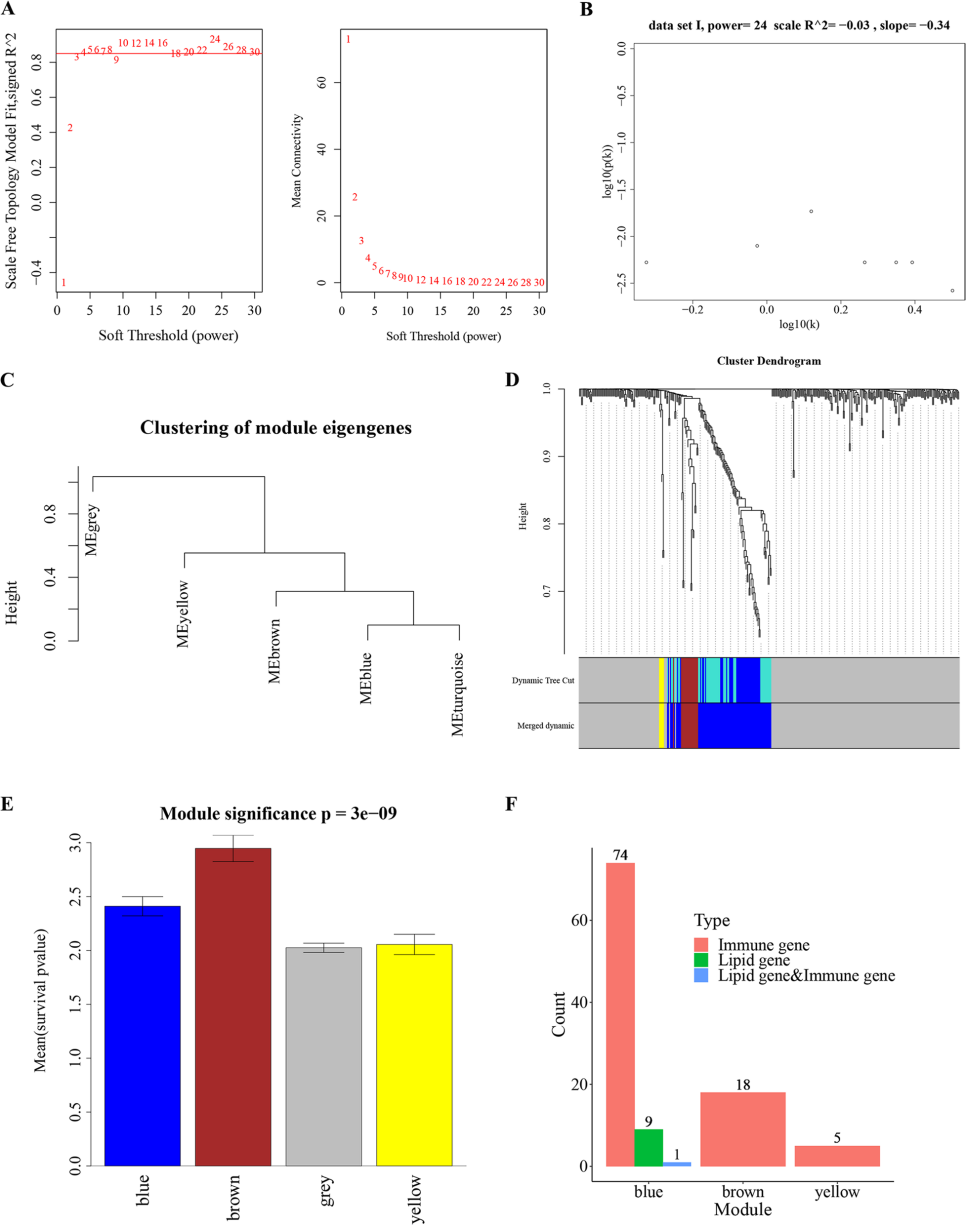
Weighted gene co-expression network analysis of lipid metabolism- and immune-related genes. **A** Determination of soft-threshold power in WGCNA. The scale-free topology index and the mean connectivity for each power value between 1 and 30 were shown. The appropriate soft-thresholding power was picked for network construction. **B** Validation of the optimal soft threshold power by the high scale-free topology R^2^ between log_10_(k) and log_10_(p(k)). k represents the connectivity between genes; and p(k) represents the probability of connectivity. **C** Hierarchical clustering tree based on WGCNA module eigengenes. Five modules were identified including grey, yellow, brown, blue, and turquoise modules. **D** The hierarchical clustering dendrogram of the eigengenes based on hierarchical clustering under optimal soft-thresholding power. Dynamic tree cut represents module divided according to clustering results; and merged dynamic represents module divided according to similarity of the module. **E** Bar plot of mean gene significance across various modules. **F** The bar plot showing the gene counts in different modules. The pink bar represents the immune-related genes; the emerald green bar represents the lipid metabolism-related genes; and the blue bar represents genes related to both immune and lipid metabolism

In the process of WGCNA, we set the β value from 1 to 30, and then calculated the corresponding correlation coefficient and gene adjacency function mean. At a β of 24, the established network was found closest to the scale-free network (Fig. 3A–B). By using this β value as the network construction parameter, we built the WCGNA model. Using the dynamic cut tree algorithm to mine modules, we set at least 5 genes in each module, and the maximum connection height was 0.99 (minModuleSize = 5, cutHeight = 0.95). As a result, 5 modules were obtained including the grey, yellow, brown, blue, and turquoise modules (Fig. 3C).

After obtaining the modules, we merged the modules with correlation coefficients greater than 0.8, and obtained 4 modules, among which the brown module had the highest significance (Fig. 3D). We extracted the genes in the modules and the *P* values for univariate analysis of these genes, based on the 'mean (*P* value)' statistical module significance (Fig. 3E). Then, we counted the gene groupings in brown, blue, and yellow modules (Fig. 3F), and found that the blue module contained genes related to lipid metabolism and immunity, while other modules only contained lipid metabolism-related genes. Therefore, the blue module was regarded as the module of interest and used for the subsequent analyses.

### Hub gene screening in significant modules

**Fig. 4:**
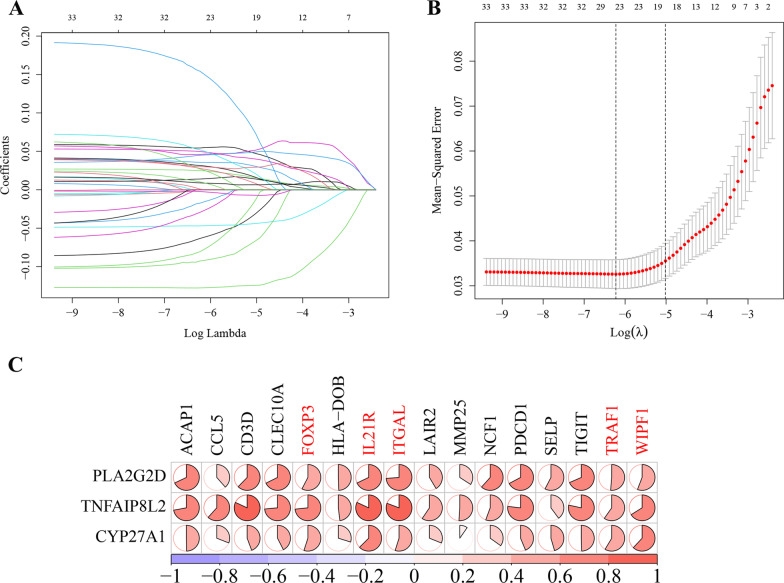
Screening hub genes by performing the LASSO analysis. **A** The LASSO coefficient profile plot of 34 DEGs. The x-axis represents the logλ, and the y-axis represents the coefficients. Every single-colored line in the plot corresponds to a predictor. **B** Cross-validation to select the optimal parameter (λ). The red dotted vertical line crosses over the optimal log λ, which corresponds to the minimum value for multivariate Cox modeling. The two dotted lines represent one standard deviation from the minimum value. **C** The correlation between 3 immune-related genes and 16 lipid metabolism-related genes

Based on the thresholds of *P*.adjust < 0.01 and |logFC|> 0.5 for significantly differentially expressed genes, a total of 34 DEGs were obtained. The LASSO regression analysis was used to remove redundant genes from the 34 DEGs (Fig. 4A–B) and obtained 19 differentially expressed genes (Table 3). The 19 genes included 16 lipid metabolism-related genes, 2 immune-related genes (PLA2G2D and TNFAIP8L2) and one common gene (CYP27A1). Figure 4C shows that 3 immune-related genes (PLA2G2D, TNFAIP8L2 and CYP27A1) were highly correlated with FOXP3, IL21R, ITGAL, TRAF1 and WIPF1 (cor > 0.5) (Fig. 4C). Therefore, these 3 immune-related genes (PLA2G2D, TNFAIP8L2 and CYP27A1) and 5 highly related lipid metabolism-related genes (FOXP3, IL21R, ITGAL, TRAF1 and WIPF1) were considered as hub genes.

**Table 3:** Differential expression analysis results of 19 module genes obtained by LASSO analysis

Gene	Type	logFC	*P*.value	*P*.adj
ACAP1	Immune gene	0.71694	1.15E−08	1.07E−07
CCL5	Immune gene	1.712164	2.35E−11	3.96E−10
CD3D	Immune gene	0.710035	0.001712	0.003596
CLEC10A	Immune gene	− 1.02433	4.69E−08	3.28E−07
CYP27A1	Lipid gene & Immune gene	− 0.86826	0.00031	0.000868
FOXP3	Immune gene	1.42025	2.37E−15	1.99E−13
HLA-DOB	Immune gene	0.895276	6.30E−11	8.83E−10
IL21R	Immune gene	0.897813	5.16E−09	5.41E−08
ITGAL	Immune gene	0.548786	0.00202	0.004099
LAIR2	Immune gene	0.876033	5.80E−15	2.44E−13
MMP25	Immune gene	0.680843	2.30E−08	1.93E−07
NCF1	Immune gene	0.597943	7.89E−06	3.68E−05
PDCD1	Immune gene	0.582185	0.001117	0.00254
PLA2G2D	Lipid gene	0.615365	0.003624	0.006349
SELP	Immune gene	− 0.769	0.00019	0.000614
TIGIT	Immune gene	1.138603	6.02E−12	1.67E−10
TNFAIP8L2	Lipid gene	0.610135	0.000215	0.000644
TRAF1	Immune gene	1.110312	7.94E−12	1.67E−10
WIPF1	Immune gene	0.994727	2.30E−09	2.76E−08

#### The relationship between the methylation levels of the 3 immune-related genes and clinical characteristics of HNSC

**Fig. 5:**
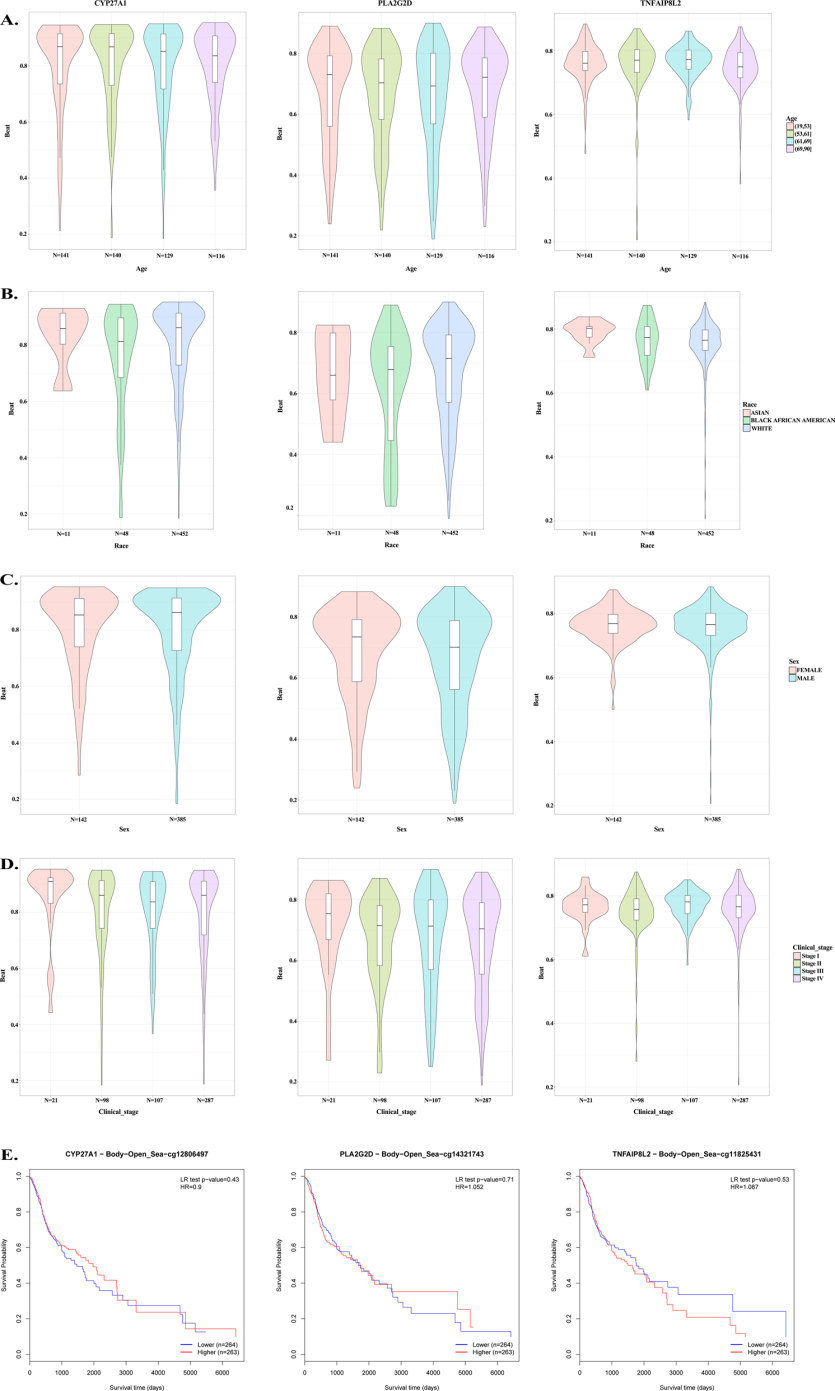
Results of DNA methylation analyses. **A** Differences in methylation levels of CYP27A1, PLA2G2D, and TNFAIP8L2 in different groups of patients divided by age. **B** Differences in methylation levels of CYP27A1, PLA2G2D, and TNFAIP8L2 in different groups of patients divided by race. **C** Differences in methylation levels of CYP27A1, PLA2G2D, and TNFAIP8L2 in different groups of patients divided by gender. **D** Differences in methylation levels of CYP27A1, PLA2G2D, and TNFAIP8L2 in different groups of patients divided clinical stage. **E** The survival curves for CYP27A1-CpG cg14321743, PLA2G2D-CpG cg11825431, and TNFAIP8L2-CpG cg12806497 in HNSC

The methylation levels of CYP27A1 were higher than that of TNFAIP8L2 and CYP27A1, irrespective of clinical feature analyzed. Figure 5A shows that the methylation level of CYP27A1 in patients younger than 61 years old was higher compared to that in patients older than 61 years and the lowest methylation level of CYP27A1 was observed in patients older than 69 years. The methylation level of PLA2G2D in patients older than 69 years was higher compared with that among patients with the age ranges 65–61 years and 61–69 years. The methylation expression level of TNFAIP8L2 in patients older than 61 years was lower compared to that of patients younger than 61 years old and the lowest methylation expression level of TNFAIP8L2 was observed in patients older than 69 years (Fig. 5).

The methylation level of CYP27A1 in African American patients was lower compared to that in Asian patients and White patients. The methylation level of PLA2G2D in White patients was higher than that in Asian and African American patients. The methylation level of TNFAIP8L2 in Asian patients was higher compared to that for African American patients and White patients (Fig. 5B).

No significant differences in the methylation level of CYP27A1 and TNFAIP8L2 were noted between female and male patients, while the methylation level of PLA2G2D in male patients was lower than that in female patients (Fig. 5C).

The highest methylation level of CYP27A1 was seen in patients with clinical stage I; while the lowest methylation expression of CYP27A1 was seen in patients with clinical stage III. The highest methylation level of PLA2G2D was seen in patients with clinical stage I; while the lowest methylation level of PLA2G2D was seen in patients of clinical stage IV. The highest methylation level of *TNFAIP8L2* was seen in patients of clinical stage III, while the lowest methylation expression of TNFAIP8L2 was seen in patients of clinical stage II (Fig. 5D).

Kaplan–Meier (Fig. 5E) revealed that methylation levels of CYP27A1 (*P* = 0.43), TNFAIP8L2 (*P* = 0.71), and CYP27A1 (*P* = 0.53) were not significantly associated with the prognosis of HNSC patients.

### Correlation between 3 immune-related hub genes and TIICs

**Fig. 6:**
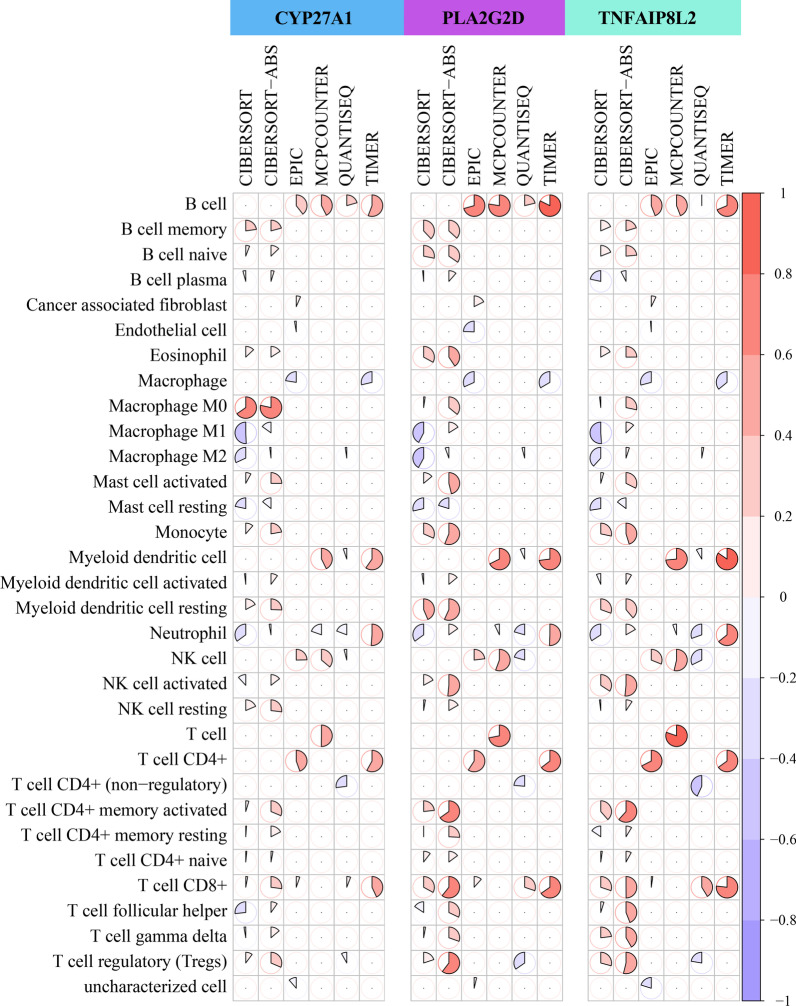
Correlation between the 3 immune-related hub genes (CYP27A1, PLA2G2D, and TNFAIP8L2) and tumor infiltrating immune cells in HNSC. Circles with red color represent a positive correlation, while circles with violet color represent a negative correlation

A negative correlation with the highest |r| value was observed between TNFAIP8L2 and Macrophage M1 (r = − 0.51, *P* = 1.56E−34), identified by the CIBERSORT method. A positive correlation with the highest |r| value was observed between TNFAIP8L2 and Myeloid dendritic cell_TIMER (r = 0.85, *P* = 2.361–138). The lowest *P* value for correlation was also observed between TNFAIP8L2 and Myeloid dendritic cell_TIMER (r = 0.85, *P* = 2.361–138) (Fig. 6, Table 4).

**Table 4:** The correlation between 3 immune-related hub genes and TIICs identified by TIMER web tool

Immune cell type_computational method	Gene	r value	*P* value	Significance
Myeloid dendritic cell_TIMER	TNFAIP8L2	0.845943402	2.36E−138	***
B cell_TIMER	PLA2G2D	0.823188557	9.29E−125	***
T cell_MCPCOUNTER	TNFAIP8L2	0.802599364	4.83E−114	***
Macrophage M0_CIBERSORT-ABS	CYP27A1	0.784330732	1.52E−105	***
B cell_MCPCOUNTER	PLA2G2D	0.774289056	3.21E−101	***
T cell CD8+ _TIMER	TNFAIP8L2	0.767609412	1.83E−98	***
Myeloid dendritic cell_MCPCOUNTER	TNFAIP8L2	0.735400667	2.25E−86	***
Myeloid dendritic cell_TIMER	PLA2G2D	0.730683202	9.42E−85	***
T cell_MCPCOUNTER	PLA2G2D	0.71933634	5.49E−81	***
B cell_EPIC	PLA2G2D	0.706689138	5.29E−77	***

Pairs with a strong significant correlation (|r|> 0.7) are listed in this table

***P < 0.001

#### The targeting relationship between immune-related hub genes and small molecule drugs

Table 5 shows the top 10 positive and negative scoring small molecules with normalized WTCS (Weighted Connectivity Score) values selected as the predicted target small molecules. The small molecule drug targeted by PLA2G2D was mepacrine. No small molecule drug was predicted for the other two immune-related hub genes (TNFAIP8L2 and CYP27A1).

**Table 5:** The significant module genes-targeted small molecule drugs predicted using the CMap database

Small molecular drugs	Cell line	Mechanism of action	Target gene	− log_10_ FDR	Normalized WTCS values
Halcinonide	HA1E	Glucocorticoid receptor agonist	NR3C1	1.7918	1.8832
GSK-1904529A	A549	IGF-1 inhibitor	IGF1R|INSR	1.6662	1.8579
SEW-2871	VCAP	Lysophospholipid receptor agonist	S1PR1	1.585	1.8371
SSR-69071	MCF7	Leukocyte elastase inhibitor	ELANE	1.4601	1.7966
Fenobam	A375	Glutamate receptor antagonist	GRM5	1.439	1.7886
BRD-K33583600	HEPG2	Guanylate cyclase activator	AKR1B1|HRH2|SIRT1|GABBR1	1.3241	1.7371
CP-55940	HCC515	Cannabinoid receptor agonist	CNR1|CNR2|GPR55	1.3225	1.7362
Dipyridamole	MDAMB231	Phosphodiesterase inhibitor	PDE5A	1.3177	1.7337
Estrone	HCC515	Estrogen receptor agonist	ESR1|ESR2	1.2876	1.7176
Ixazomib	HEPG2	Proteasome inhibitor	PSMB1	1.2793	1.7129
I-BET-762	OCILY3	Bromodomain inhibitor	BRD2|BRD3|BRD4|APOA1	0.9565	− 1.6703
SB-590885	HCC515	RAF inhibitor	BRAF	0.9565	− 1.686
Methylnorlichexanthone	HA1E	Aurora kinase inhibitor|PIM inhibitor|VEGFR inhibitor	AURKB|PIM1	0.9565	− 1.6863
CHIR-99021	HA1E	GSK inhibitor	CDK1|GSK3A|GSK3B|MAPK1	0.9565	− 1.6981
Enalapril	HA1E	ACE inhibitor	ACE	0.9565	− 1.7012
XMD-1150	OCILY3	LRRK inhibitor	LRRK2	0.9565	− 1.7161
Nimesulide	HCC515	Cyclooxygenase inhibitor	PTGS2|LTF|PLA2G2E|PTGS1	0.9565	− 1.7362
Olopatadine	PC3	Histamine receptor antagonist	HRH1	0.9565	− 1.7392
Oxalomalic-acid	A549	Isocitrate dehydrogenase inhibitor	ACO1|IDH1	0.9565	− 1.7631
PTB1	HEPG2	AMPK activator	PTPN1	0.9565	− 1.8713
Mepacrine	THP1	NFKB inhibitor|Cytokine production inhibitor|p53 activator	PLA2G2A	0.0953	0.9348
Mepacrine	NCIH508	NFKB inhibitor|Cytokine production inhibitor|p53 activator	PLA2G2A	0.0853	0.9055
Mepacrine	HUVEC	NFKB inhibitor|Cytokine production inhibitor|p53 activator	PLA2G2A	0.0114	0.7241
Mepacrine	H1299	NFKB inhibitor|Cytokine production inhibitor|p53 activator	PLA2G2A	0.0058	0.6972
Mepacrine	MCF10A	NFKB inhibitor|Cytokine production inhibitor|p53 activator	PLA2G2A	0	0.6437
Mepacrine	MDAMB231	NFKB inhibitor|Cytokine production inhibitor|p53 activator	PLA2G2A	0.0794	− 0.9696
Mepacrine	NCIH596	NFKB inhibitor|Cytokine production inhibitor|p53 activator	PLA2G2A	0.0983	− 1.0372
Mepacrine	A375	NFKB inhibitor|Cytokine production inhibitor|p53 activator	PLA2G2A	0.2388	− 1.259
Mepacrine	NCIH2073	NFKB inhibitor|Cytokine production inhibitor|p53 activator	PLA2G2A	0.2899	− 1.3061
Mepacrine	HCC515	NFKB inhibitor|Cytokine production inhibitor|p53 activator	PLA2G2A	0.4515	− 1.4688

### The expression pattern of hub genes

**Fig. 7:**
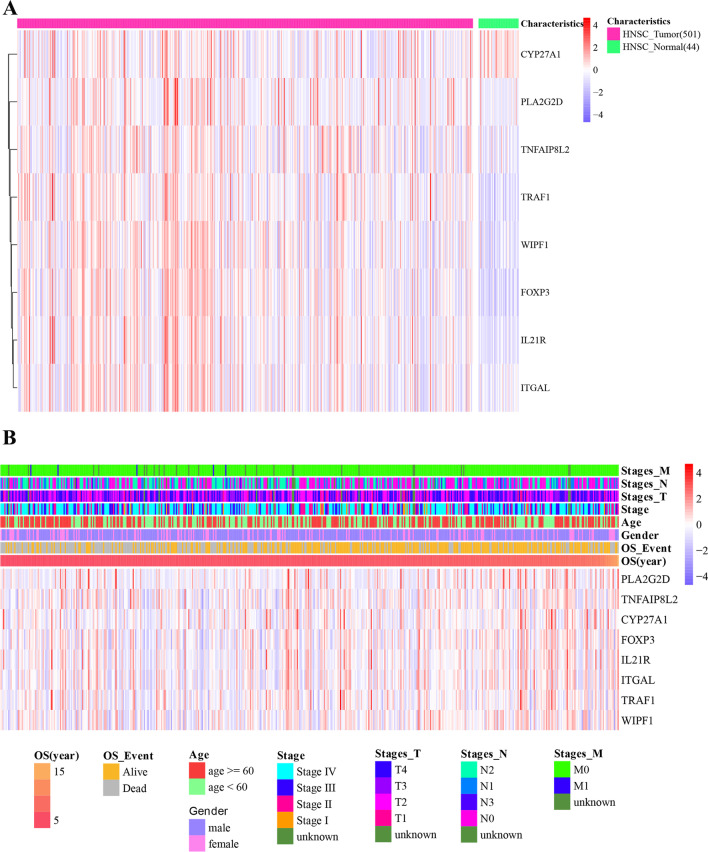
Expression levels of hub genes in **A** HNSC tumor samples and head and neck normal samples; **B** groups divided by different clinical features (T stage, N stage, M stage, clinical stage, age, OS_event, and OS_year). The red color represents upregulation, while the violet color represents downregulation. The darker colour represents the larger |logFC| values

A heatmap was used to show the expression values of the eight hub genes in the HNSC tumor and normal samples. CYP27A1 was downregulated in HNSC tumor samples, while the other genes were upregulated in HNSC tumor samples (Fig. 7A) A heatmap (Fig. 7B) depicted the expression levels related to different clinical features.

**Fig. 8:**
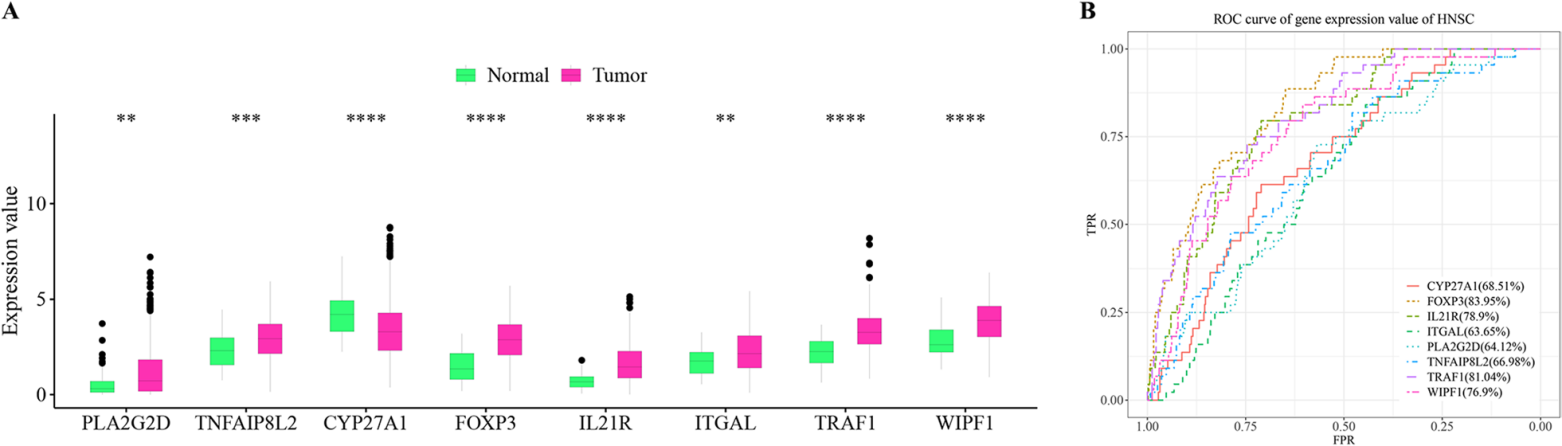
Differential expression and diagnostic values of hub genes. **A** The expression pattern of hub genes (PLA2G2D, TNFAIP8L2, CYP27A1, FOXP3, IL21R, ITGAL, TRAF1, and WIPF1) in HNSC tumor samples and healthy control samples. **P < = 0.01, ***P < = 0.001, ****P < = 0.0001. **B** The ROC curves showing the diagnostic values of hub genes (CYP27A1 (AUC = 68.51%), FOXP3 (AUC = 83.95%), IL21R (78.9%), ITGAL (63.65%), PLA2G2D (64.12%), TNFAIP8L2 (66.98%), TRAF1 (81.04%), WIPF1 (76.9%)). The x axis represents false positive rate (FPR), and y axis represents true positive rate (TPR)

The hub genes were significantly dysregulated between tumor and healthy control samples (Fig. 8A). The overall expression level of CYP27A1 was higher than that of the other hub genes. The ROC analysis showed five lipid metabolism-related hub genes (FOXP3, IL21R, ITGAL, TRAF1 and WIPF1) had higher predictive values than three immune-related hub genes (PLA2G2D, TNFAIP8L2 and CYP27A1) (Fig. 8B).

### Grouping tumor samples of HNSC based on multivariate analysis

**Fig. 9:**
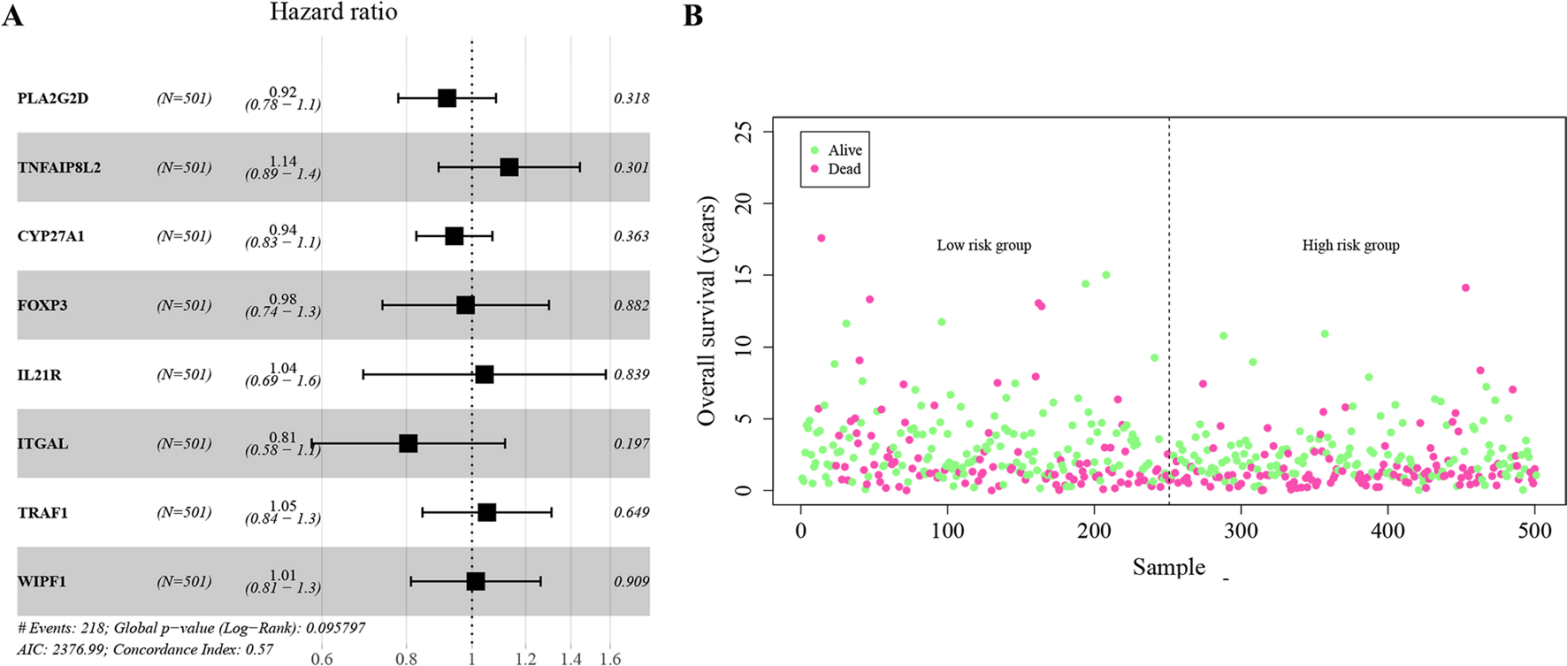
**A** Forest plot with hazard ratio (HR) for the 8 hub genes of the multivariable model. **B** The gene expression levels of the hub genes associated with “survival state” in low risk group and high risk group. The green dots represent the “survival state” to be alive, while the red dots represent the “survival state” to be dead

**Fig. 10:**
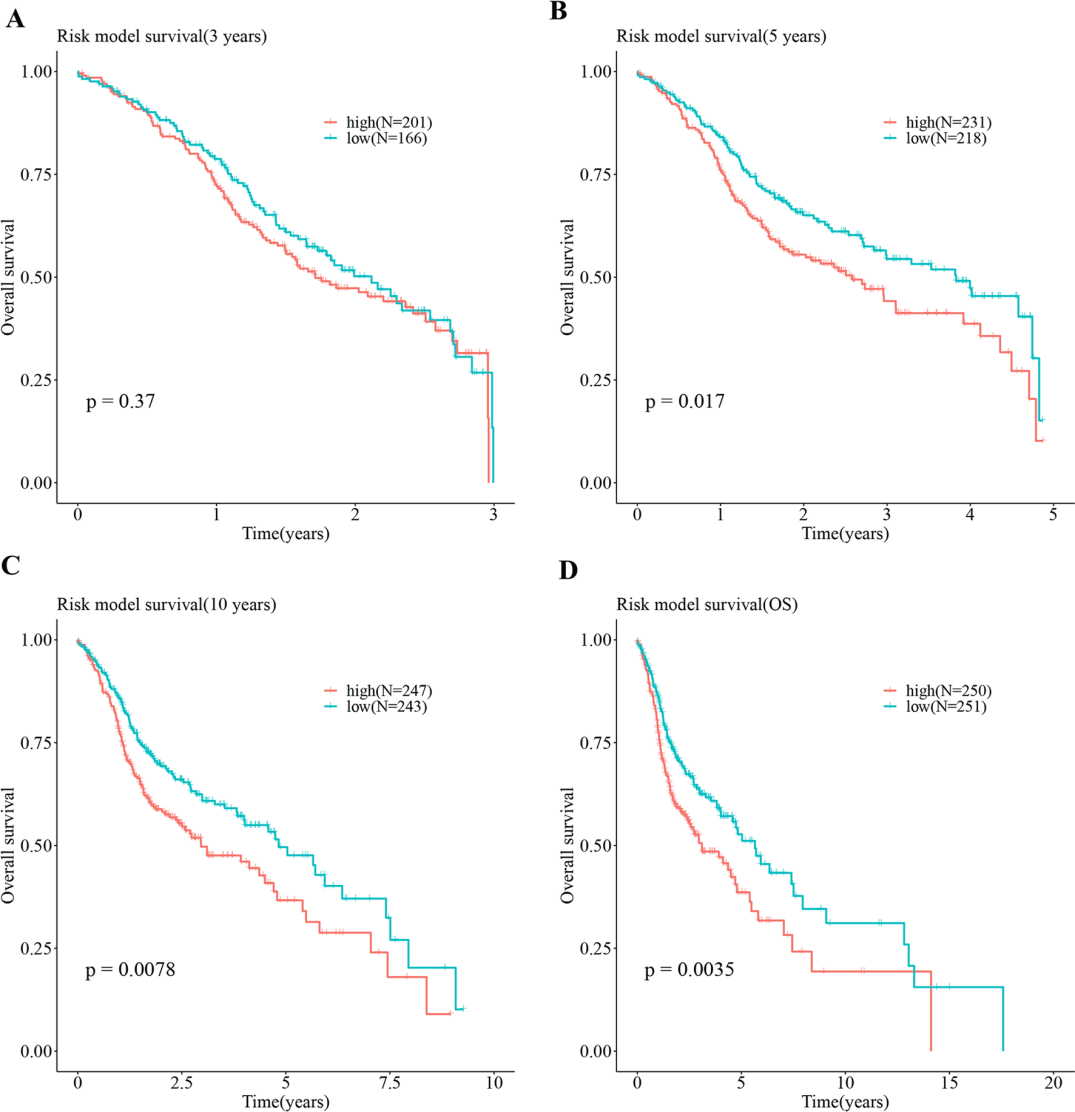
The difference in survival probability between the high-risk and low-risk groups at the different time periods (3 year (**A**), 5 year (**B**), 10 year (**C**), and 20 year (**D**)). The x axis represents time points (year), and the y axis represents the overall survival

**Fig. 11:**
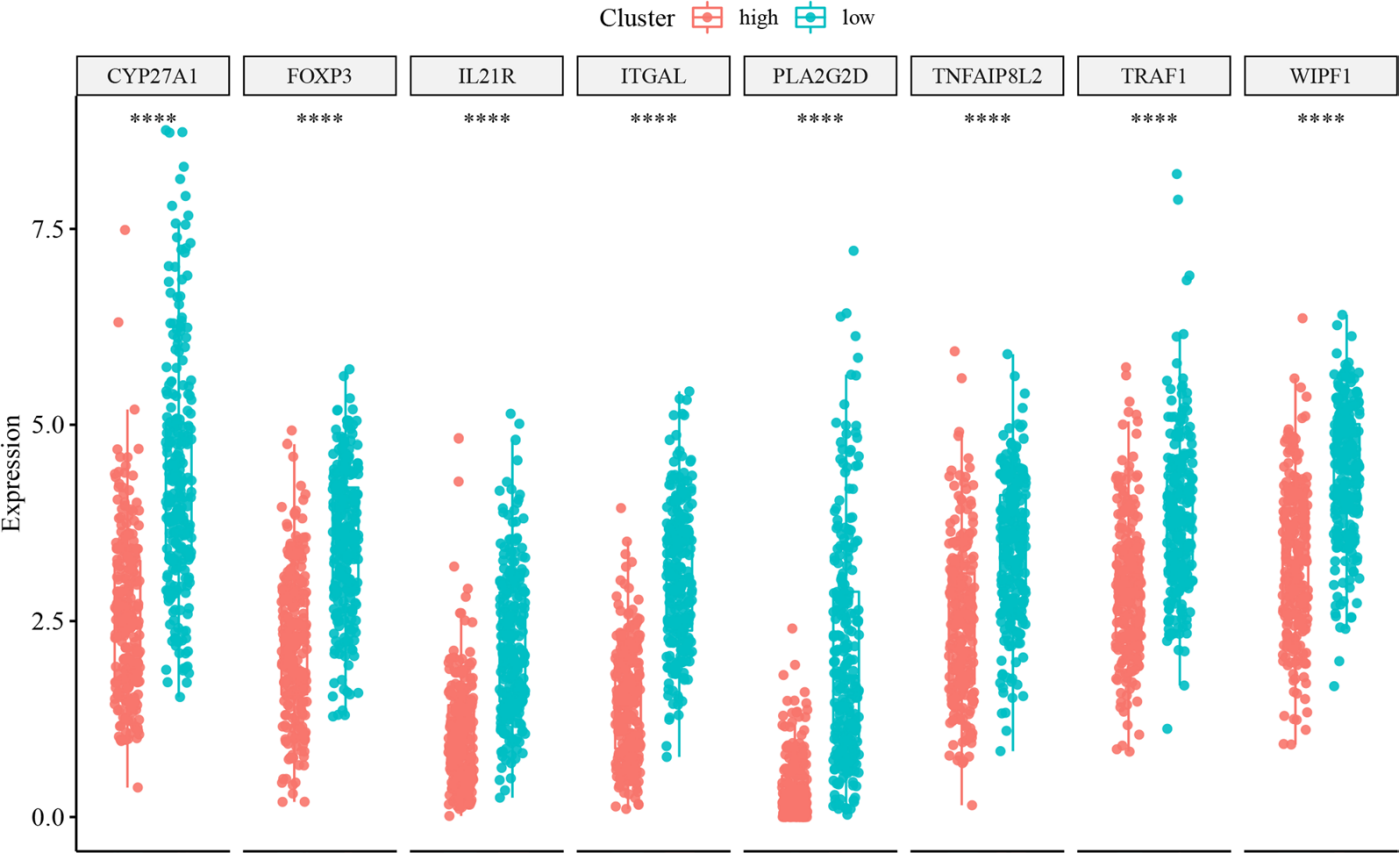
Dot plot showing the expression levels of Hub genes in high risk and low risk groups. The red color represents the high risk patients cluster, and the green color represents the low risk patients cluster

We extracted the expression values of the hub genes in the tumor case samples, and established a Cox-PH model based on the clinical characteristics of OS and OS_Event for multivariate analysis. The results showed that the expression patterns of 8 hub genes were significantly related with the overall survival outcomes of HNSC patients (Fig. 9A). The samples were divided into high-risk and low-risk groups based on the median of their risk scores. The number of non-surviving samples in the high-risk group was higher than that in the low-risk group (Fig. 9B). The survival probability of high-risk and low-risk groups within 3 years was not significantly different (*P* = 0.37) (Fig. 10A). However, there were significant differences between the high-risk group and the low-risk group in the other three time periods (i.e., 5 year (*P* = 0.017), 10 year (*P* = 0.0078), and 20 year (*P* = 0.0035), showing that the survival rate of the high-risk group was significantly lower than that of the low-risk group (Fig. 10B–D). These findings indicated that as the survival time of HNSC patients increases, lipid metabolism-related genes and immune-related genes have a greater impact on survival. The 8 hub genes were expressed differentially between high- and low-risk groups, with the high-risk group expressing each hub gene at a lower level compared with the low-risk group (Fig. 11).

### Association of clinical characteristics and risk groups with survival

**Fig. 12:**
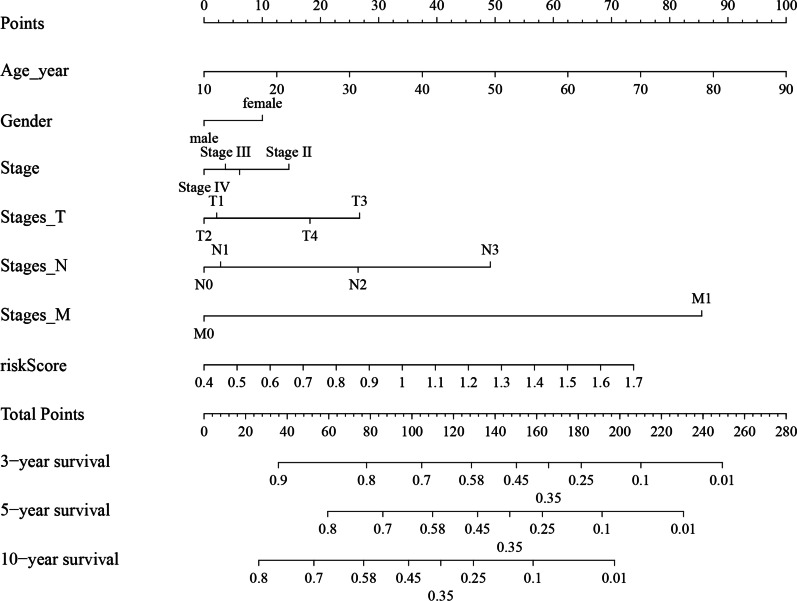
Nomogram for prediction of 3-year, 5-year, and 10-year overall survival based on independent risk factors (age_year, gender, stage, T stage, N stage, M stage, and risk score) for HNSC patients. The survival probability was estimated by calculating the total points

**Fig. 13:**
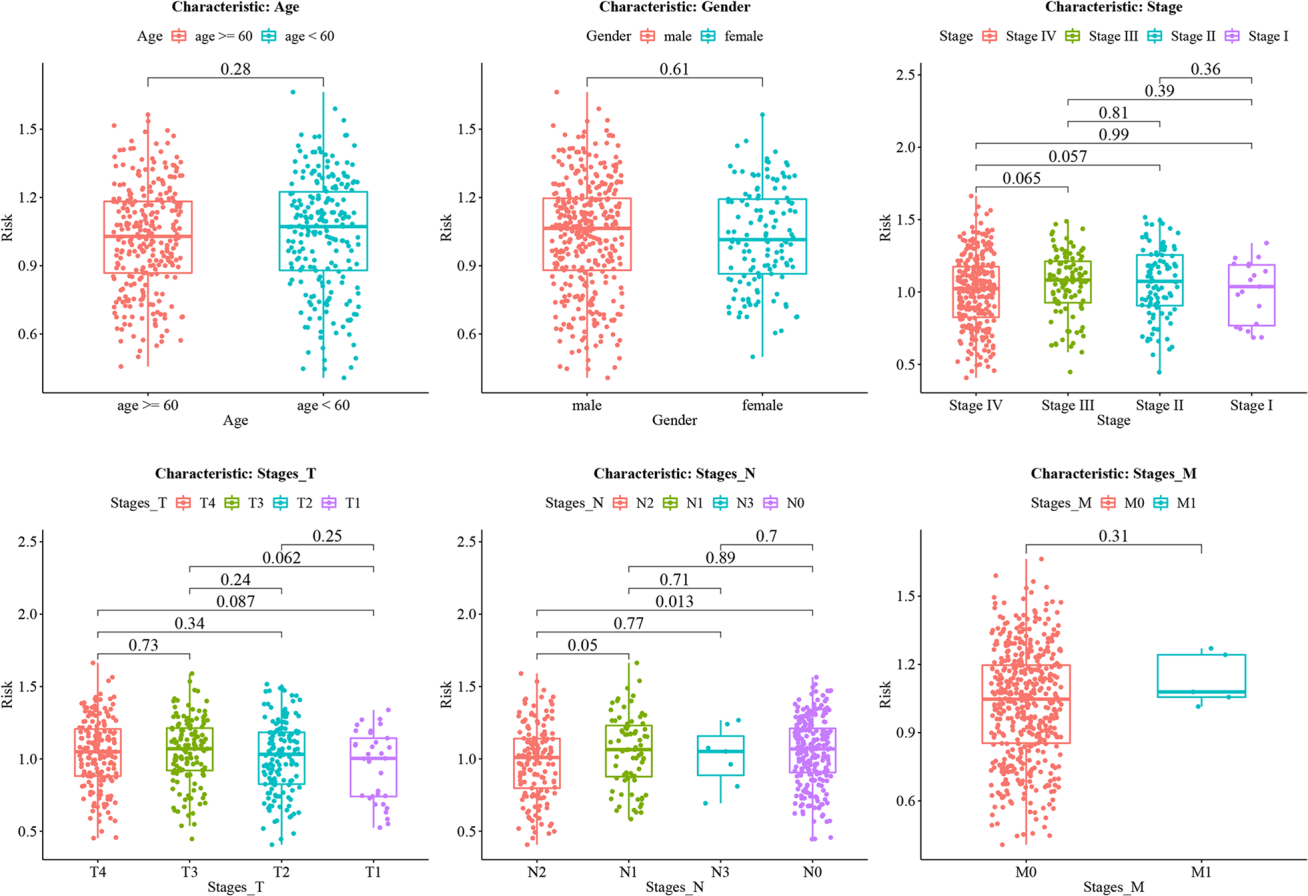
Relationship between survival risk and different clinicopathological features (e.g., age, gender, stage, T stage, N stage, and M stage). HNSC tumor patients were divided into different groups based on a specific clinicopathological feature, and box plot was drawn to show the difference of survival risk between different groups

**Fig. 14:**
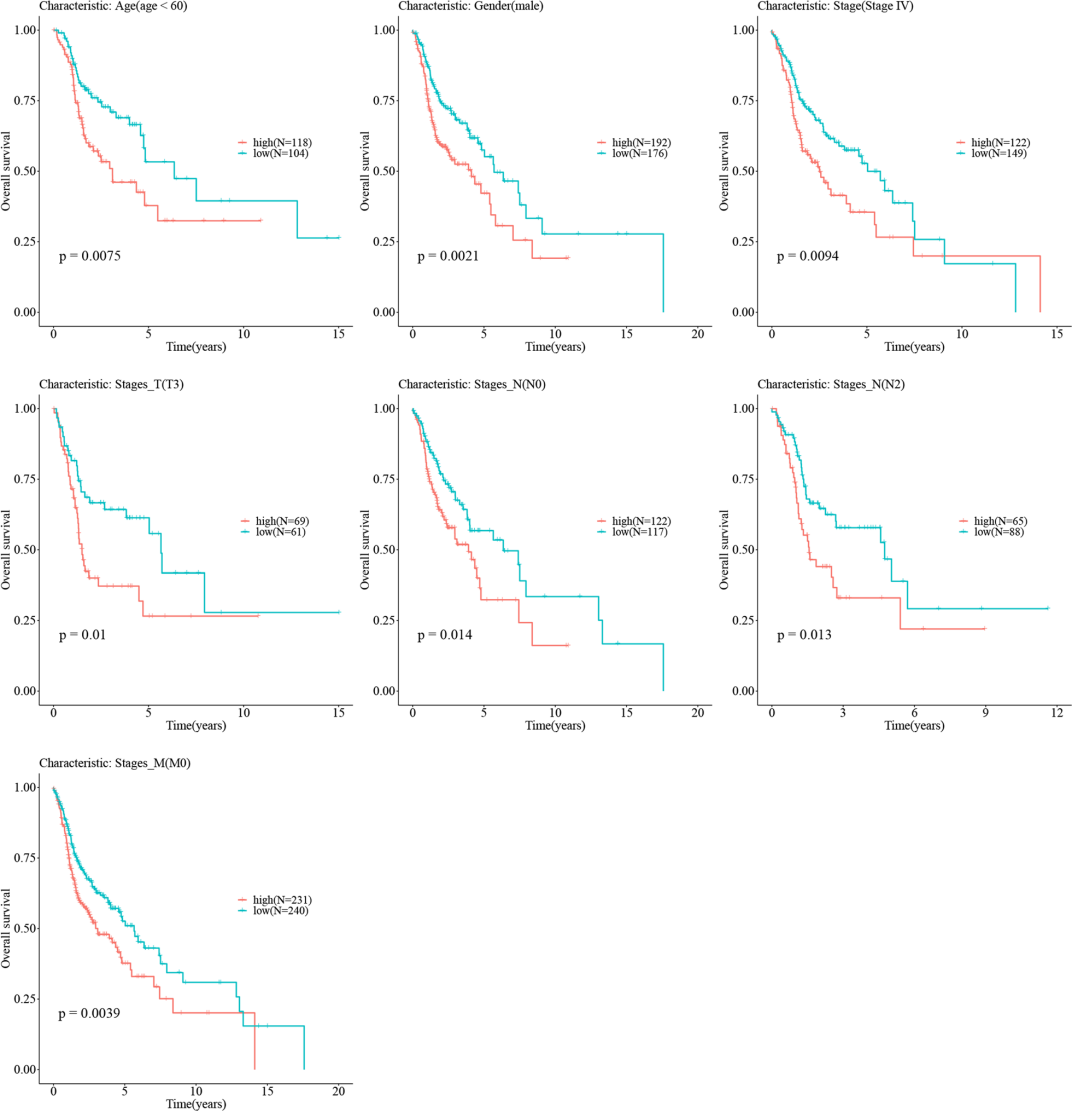
Kaplan–Meier plot showing the relationship between high-risk samples and low-risk samples and survival in different clinical feature groups. The red curve represents the HNSC patients with high risk, while the green curve represents the HNSC patients with low risk. The x axis is time points (year), and the y axis is the overall survival probability

A nomogram of 467 samples was plotted to demonstrate the effect of different clinical characteristics and risk scores on survival (Fig. 12). Figure 13 shows the differences in risk scores among subjects grouped by clinical characteristics. Figure 14 shows that the patients with clinical characteristics age < 60, gender = male, stage = (IV, T3, N0, N2)) showed significant differences in terms of overall survival outcomes between high- and low- risk groups, and the survival probability of patients in the high risk group was lower than that in the low risk group.

### The somatic mutation of hub genes

**Fig. 15:**
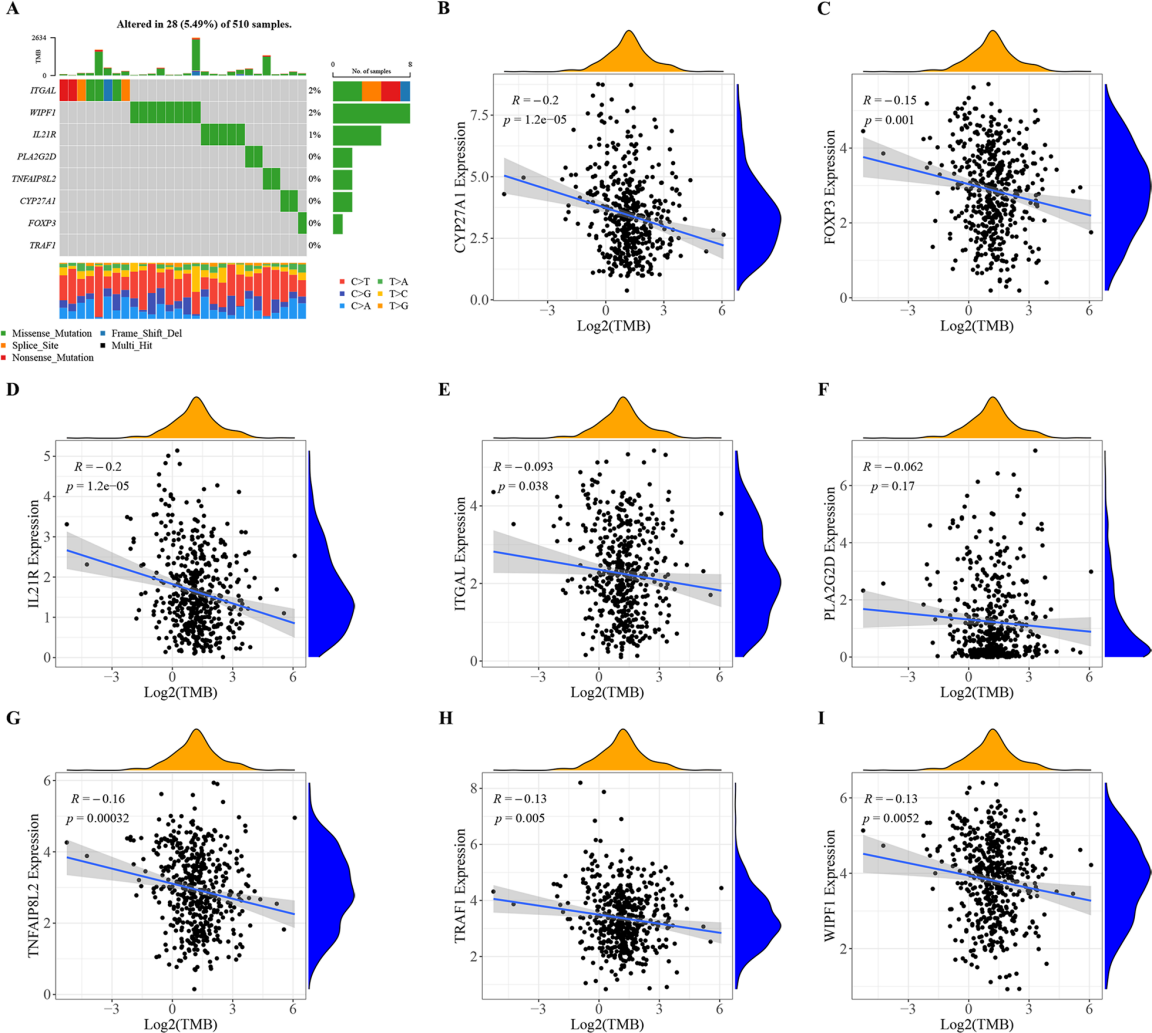
Somatic mutation analysis of Hub genes. **A** Mutation of hub genes in tumor samples. In the figure, the horizontal axis represents samples, and the vertical axis represents genes. The scale value on the right side of the picture refers to the percentage of samples with gene mutation in the total sample. The gray square indicates that the sample has not been mutated. Other colors are variant samples. The bar graph above the picture depicts the number and variant types of all variant genes in each sample, and the bar graph on the right side of the picture depicts the number of mutated samples in the current gene. The bar chart below the picture depicts the changes of the bases in each sample. **B**–**I** Scatter plots showed the correlation between hub genes and TMB. The x axis represents the log_2_TMB, and the y axis represents the expression value of each hub gene. “R” indicates the correlation coefficient value, and *P* value indicates the statistical significance

ITGAL and WIPF1 were the most mutated in tumor samples (Fig. 15A). TNFAIP8L2, CYP27A1, FOXP3, IL21R, ITGAL, TRAF1, WIPF1 and TMB were significantly correlated; however, PLA2G2D was not significantly correlated with TMB (Figs. 15B, 13I).

### Pathway network analysis of hub genes

**Fig. 16:**
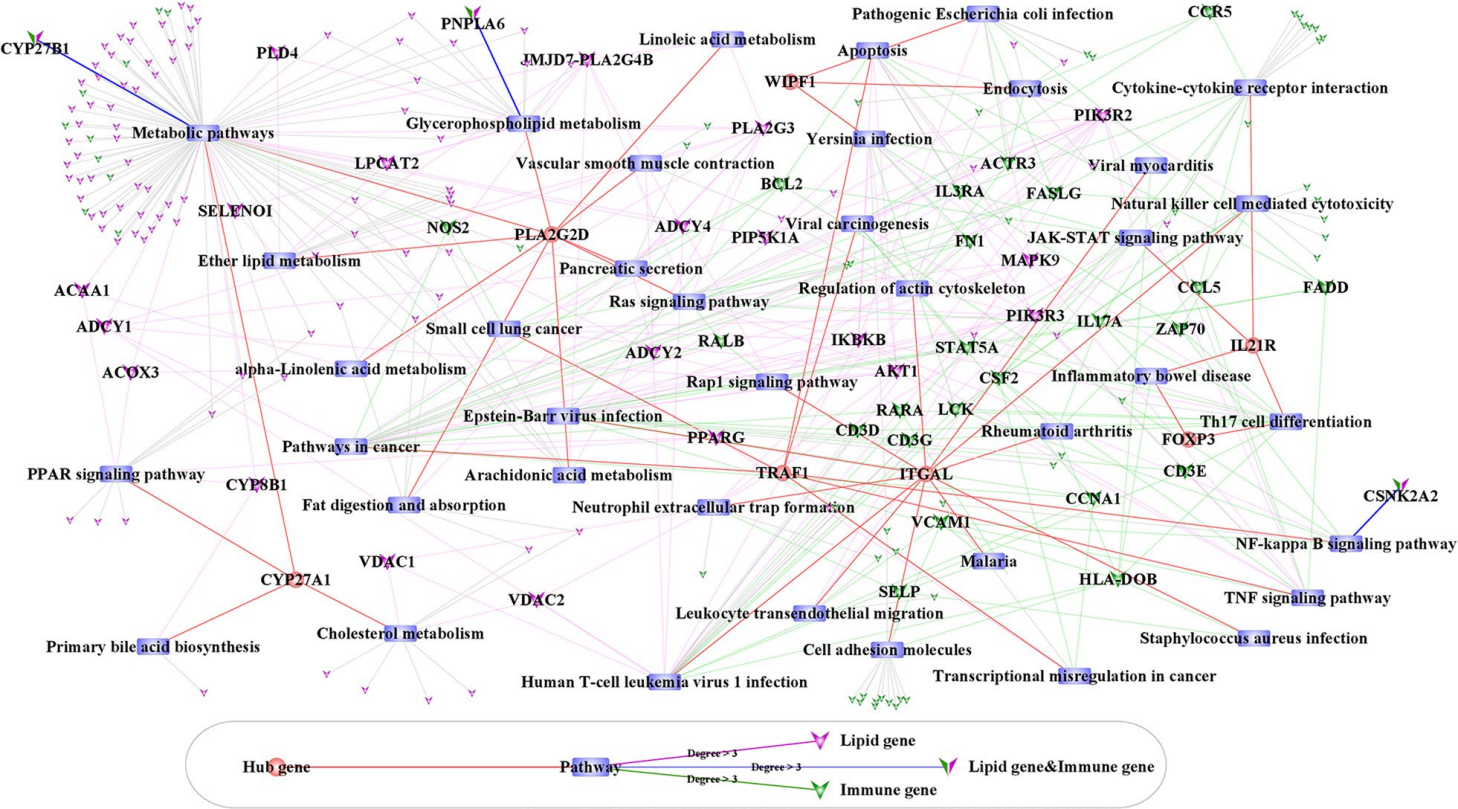
Hub gene-Pathway-survival genes network. The network contains 241 interacting nodes (7 Hub genes, 39 Pathways, 115 Lipid genes, 77 Immune genes and 3 Lipid genes & Immune genes) and 463 interacting nodes and edges. The circle nodes represent hub genes, rectangular nodes represent pathway. The red arrow nodes represent the lipid metabolism-related genes; the green arrow nodes represent the immune-related genes; and the arrow nodes with half green and half red represent the genes related to both lipid metabolism and immune

Figure 16 shows that CYP27A1 and CYP27B1 were involved in metabolic pathways. Both CYP27A1 and CYP27B1 are genes related to lipid metabolism and immunity. The lipid metabolism-related genes CYP27A1, ACOX3, ADCY1 and ACAA1, are also involved in the regulatory pathway PPAR signaling pathway. The hub genes co-regulate cancer-related pathways by directly or indirectly interacting with other lipid metabolism genes or immune-related genes.

## Discussion

The main findings of the current research identified 3 immune-related genes (PLA2G2D, TNFAIP8L2, and CYP27A1) which are likely to play highly significant roles in lipid metabolism-mediated tumor immunity regulation in HNSC. PLA2G2D (Phospholipase A2, Group IID) encodes the secreted phospholipase A2 enzyme that lyses glycerophospholipids to yield lysophospholipids and free fatty acids [36]. PLA2G2A is induced and secreted by immune cells including dendritic cells and macrophages when stimulated by proinflammatory mediators, and also results in augmented production of proinflammatory lipid and cytokines by acting on phospholipids [37]. Dendritic cells in lymphoid tissues abundantly express PLA2G2D, which regulates the steady-state levels of anti-inflammatory lipids in order to resolve Th1 immune response [36]. Chronic inflammation induced by PLA2G2A could contribute to the promotion of carcinogenesis. Miki et al. found that the overexpression of PLA2G2D suppressed anti-tumor immunity by increasing tumor-promoting M2-like macrophages and decreasing tumor-suppressing M1-like macrophages and cytotoxic T cells in skin carcinoma [36]. Contrary to the findings of the current study, another study reported that PLA2G2D expression was positively correlated with immune infiltration and indicated better prognosis in HNSC patients [6]. The knockdown of PLA2G6 (Phospholipase A2 Group VI) was found to suppress the progression of melanoma by affecting the phenotypes of melanoma cells, inhibiting cell proliferation, migration, and invasion, promoting tumor cell apoptosis [38]. To our knowledge, experimental research investigating the influence of PLA2G2D on the phenotypes of HNSC cells is lacking. Of note, the small molecule drug targeted by PLA2G2D mepacrine (also named as quinacrine), which was originally used as an anti-malarial drug has been recently repurposed as an anticancer agent in treating ovarian cancer, gynecologic, breast cancer, and colon cancer [39, 40]. Mepacrine was also found to inhibit cell viability and clonogenic survival, as well as promote apoptosis of five types of HNSC cell lines (CAL27, SCC040, FaDu, SCC47 and VU147) [41]. Mepacrine induced TP53 mRNA and protein expression, increased TP53 reporter activity and p21 protein expression, and induced growth inhibition in the wild-type TP53 HNSC cell lines [42].

TNFAIP8L2 (TNF Alpha Induced Protein 8 Like 2; also named as TIPE2) is a lipid transfer protein and can inhibit lipid biosynthesis pathways by negatively regulating lipid biosynthesis-related gene signatures [43]. TNFAIP8L2 acts as a negative regulator of innate and adaptive immunity by inhibiting the function of Toll-like receptor and T-cell receptor [44]. TNFAIP8L2 was found to positively regulate and enhance the anti-tumor immune response in head and neck cancer [45]. A previous research comprehensively assessed the expression and prognosis of TNFAIPs family members in head and neck cancer and found a positive correlation between TNFAIP8 and tumor infiltrating immune cells macrophage, neutrophil, CD8+ T cell, CD4+ T cell, and dendritic cell) [45]. In addition, the overexpression of TNFAIP8L2 predicted improved overall survival in head and neck cancer patients [45]. TNFAIP8L2 was also found significantly associated with cancer stem cell index, indicating its potential role as a novel immune checkpoint gene for the immunotherapy of cancers [46]. The transfection of TNFAIP8L2 in hepatocellular carcinoma (HCC) cell lines markedly inhibited tumor cell growth, migration and invasion in vitro [47], but research investigating the effects of TNFAIP8L2 upregulation on the phenotypes of HNSC cells is lacking.

CYP27A1 (Cytochrome P450 Family 27 Subfamily A Member 1) is a key enzyme involved in the process of bile acid synthesis and involved in regulating cellular cholesterol homeostasis. Mutations in CYP27A1 result in reduced bile acid synthesis, increased production of cholestanol, and subsequently cholestanol accumulation [48]. The dysregulation of CYP27A1 expression has been found to be a prognostic biomarker in breast cancer [49], prostate cancer [50], and ovarian cancer [51]. CYP27A1 was found to be highly expressed in myeloid immune cells and macrophages and played a pro-tumorigenic role in breast cancer by impairing T cell expansion [52]. However, the current research indicated that a high expression of CYP27A1 indicated the low risk of death in HNSC. In agreement, CYP27A1 was found to inhibit proliferation and migration of clear cell renal cell carcinoma by activating the LXRs/ABCA1 pathway [53]. Experimental research investigating the involvement and regulatory roles of CYP27A1 in the phenotypes of HNSC tumor cells is warranted.

The current study showed several immune cell-related signaling pathways were involved in lipid metabolism-mediated tumor immunity in HNSC, including T cell receptor signaling, Th17 cell differentiation, and natural killer (NK) cell mediated cytotoxicity. Several studies have highlighted these relationships. Modulating fatty acid metabolism can enhance CD8 T-cell memory generation, thereby further amplifying the anti-tumor immunity [54]. A high content of cholesterol in tumors inhibits anti-tumor immunity by upregulating immune checkpoint genes and further inducing CD8+ T Cell exhaustion [55]. The cholesterol esterification enzyme acetyl-CoA acetyltransferase (ACAT1) knockout in CD8+ T cells was found to downregulate membrane cholesterol and improve T cell receptor clustering and signaling, enhancing CD8+ T cell function and anti-tumor immunity in mouse tumor models [56]. Th17 lymphocytes express a proinflammatory phenotype by secreting cytokines (e.g., IL-10, IL-17, IFN-γ, and IL-22) [57] and the Th17/Treg balance relies heavily on fatty acid metabolism [58]. Short-chain fatty acids (SCFAs) activate and promote the differentiation of Th17 lymphocytes into T regulatory (Treg) cells, which play immunosuppressive functions in the tumor microenvironment [57]. The innate lymphoid cells-NK cells play a crucial role in preventing tumor metastasis [59]. NK cells in the lipid-rich environment are found to be immature and defective in the ability to lyse target tumor cells [60]. The cytotoxicity of NK cells is impaired by lipid accumulation in cancer patients, which contributes to an immunosuppressive tumor microenvironment [60]. Although these immune-related pathways have been implicated in lipid metabolism-mediated cancer biology, evidence in the context of head and neck cancer is lacking and merits future investigation.

This study utilized a computational biology approach to identify candidate immune-related genes and pathways involved in imbalanced lipid metabolic homeostasis-mediated tumor immunity in HNSC. Using a multi-level approach, the most significant candidates were identified, and the findings provide a theoretical foundation for future discovery of lipid metabolism-targeting therapies in HNSC. The candidate genes and mechanisms identified in this study need to be validated by designing relevant experiments. We suggest that co-culture environment models of lipid laden-HNSC cells and immune cells be established, and the genetic reciprocal interactions between HNSC cells and immune cells be examined. In vivo experiments could be also designed to observe the effects of lipid metabolism alterations on the immune function of tumor-bearing animal models. The current study was mainly focused on the effects of abnormal lipid metabolism on individual tumor infiltrating immune cells, which is far from sufficient as a particular lipid metabolic pathway might produce contradictory results for different types of immune cells [61]. Therefore, specific mechanisms underpinning lipid metabolic homeostasis imbalance in HNSC tumor immune regulation merit comprehensive research.

## Conclusion

The current research identified immune genes PLA2G2D, TNFAIP8L2, and CYP27A1 and immune cells-related pathways T cell receptor signaling, Th17 cell differentiation, and NK cell mediated cytotoxicity as the primary candidates involved in lipid metabolism-mediated tumor immunity in HNSC. These may comprise promising therapeutic biomarkers and targets for modulation in the field of cancer immunotherapy for head and neck cancer.

## Data Availability

The data analyzed during the current study are available in the TCGA database with the accession numbers TCGA-HNSC. The original contributions presented in the study are included in the article, further inquiries can be directed to the corresponding author.

## References

[1] Bian X, Liu R, Meng Y, Xing D, Xu D, Lu Z (2021). Lipid metabolism and cancer. J Exp Med.

[2] Santos CR, Schulze A (2012). Lipid metabolism in cancer. FEBS J.

[3] Liu X, Zhang P, Xu J, Lv G, Li Y (2022). Lipid metabolism in tumor microenvironment: novel therapeutic targets. Cancer Cell Int.

[4] Broadfield LA, Pane AA, Talebi A, Swinnen JV, Fendt S-M (2021). Lipid metabolism in cancer: new perspectives and emerging mechanisms. Dev Cell.

[5] Klein JD, Grandis JR (2010). The molecular pathogenesis of head and neck cancer. Cancer Biol Ther.

[6] Xiong Y, Si Y, Feng Y, Zhuo S, Cui B, Zhang Z (2021). Prognostic value of lipid metabolism-related genes in head and neck squamous cell carcinoma. Immun Inflamm Dis.

[7] Albakri MM, Huang SC-C, Tashkandi HN, Sieg SF (2022). Fatty acids secreted from head and neck cancer induce M2-like macrophages. J Leukoc Biol.

[8] Newton HS, Chimote AA, Arnold MJ, Wise-Draper TM, Conforti L (2021). Targeted knockdown of the adenosine A2A receptor by lipid NPs rescues the chemotaxis of head and neck cancer memory T cells. Mol Ther-Methods Clin Dev.

[9] Jensen MA, Ferretti V, Grossman RL, Staudt LM (2017). The NCI genomic data commons as an engine for precision medicine. Blood J Am Soc Hematol.

[10] Katsonis P, Koire A, Wilson SJ, Hsu T-K, Lua RC, Wilkins AD (2014). Single nucleotide variations: biological impact and theoretical interpretation. Protein Sci.

[11] Kanehisa M, Goto S (2000). KEGG: kyoto encyclopedia of genes and genomes. Nucleic Acids Res.

[12] Liberzon A, Subramanian A, Pinchback R, Thorvaldsdóttir H, Tamayo P, Mesirov JP (2011). Molecular signatures database (MSigDB) 3.0. Bioinformatics.

[13] Wan J, Qian S-B (2014). TISdb: a database for alternative translation initiation in mammalian cells. Nucleic Acids Res.

[14] Hänzelmann S, Castelo R, Guinney J (2013). GSVA: gene set variation analysis for microarray and RNA-seq data. BMC Bioinf.

[15] Smyth GK. Limma: linear models for microarray data. In: Bioinformatics and computational biology solutions using R and Bioconductor. Springer; 2005. p. 397–420.

[16] Anders S, Huber W. Differential expression analysis for sequence count data. Nat Preced. 2010;1–1. 10.1186/gb-2010-11-10-r106 PMC3218662 20979621

[17] Therneau TM, Lumley T (2015). Package ‘survival’. R Top Doc.

[18] Harrell FE. Cox proportional hazards regression model. In: Regression modeling strategies. Springer; 2015. p. 475–519.

[19] Langfelder P, Horvath S (2008). WGCNA: an R package for weighted correlation network analysis. BMC Bioinf.

[20] Shuai M, Chen X. Algorithm optimization for weighted gene co-expression network analysis: accelerating the calculation of Topology Overlap Matrices with OpenMP and SQLite. bioRxiv. 2021.

[21] Weppler S, Schinkel C, Kirkby C, Smith W (2020). Lasso logistic regression to derive workflow-specific algorithm performance requirements as demonstrated for head and neck cancer deformable image registration in adaptive radiation therapy. Phys Med Biol.

[22] Fawcett T (2006). An introduction to ROC analysis. Pattern Recognit Lett.

[23] Modhukur V, Iljasenko T, Metsalu T, Lokk K, Laisk-Podar T, Vilo J (2018). MethSurv: a web tool to perform multivariable survival analysis using DNA methylation data. Epigenomics.

[24] Anuraga G, Wang W-J, Phan NN, An Ton NT, Ta HDK, Berenice Prayugo F (2021). Potential prognostic biomarkers of NIMA (Never in Mitosis, Gene A)-Related Kinase (NEK) family members in breast cancer. J Pers Med.

[25] Song Y, Ma R (2022). Identifying the potential roles of PBX4 in human cancers based on integrative analysis. Biomolecules.

[26] Li T, Fu J, Zeng Z, Cohen D, Li J, Chen Q (2020). TIMER2. 0 for analysis of tumor-infiltrating immune cells. Nucleic Acids Res.

[27] Kao T-J, Wu C-C, Phan NN, Liu Y-H, Ta HDK, Anuraga G (2021). Prognoses and genomic analyses of proteasome 26S subunit, ATPase (PSMC) family genes in clinical breast cancer. Aging.

[28] Laham AJ, El-Awady R, Lebrun J-J, Ayad MS (2022). A bioinformatics evaluation of the role of dual-specificity tyrosine-regulated kinases in colorectal cancer. Cancers.

[29] Benesty J, Chen J, Huang Y, Cohen I. Pearson correlation coefficient. In: Noise reduction in speech processing. Springer; 2009. p. 1–4.

[30] Musa A, Ghoraie LS, Zhang S-D, Glazko G, Yli-Harja O, Dehmer M (2018). A review of connectivity map and computational approaches in pharmacogenomics. Brief Bioinform.

[31] Subramanian A, Narayan R, Corsello SM, Peck DD, Natoli TE, Lu X (2017). A next generation connectivity map: L1000 platform and the first 1,000,000 profiles. Cell.

[32] Wang C-Y, Chiao C-C, Phan NN, Li C-Y, Sun Z-D, Jiang J-Z (2020). Gene signatures and potential therapeutic targets of amino acid metabolism in estrogen receptor-positive breast cancer. Am J Cancer Res.

[33] Harrell FE, Harrell MFE, Hmisc D (2017). Package ‘rms’. Vanderbilt Univ.

[34] Kristensen K, Nielsen A, Berg CW, Skaug H, Bell B. TMB: automatic differentiation and Laplace approximation. ArXiv Prepr ArXiv150900660. 2015.

[35] Smoot ME, Ono K, Ruscheinski J, Wang P-L, Ideker T (2011). Cytoscape 2.8: new features for data integration and network visualization. Bioinformatics.

[36] Miki Y, Kidoguchi Y, Sato M, Taketomi Y, Taya C, Muramatsu K (2016). Dual roles of group IID phospholipase A2 in inflammation and cancer. J Biol Chem.

[37] Murakami M, Yamamoto K, Miki Y, Murase R, Sato H, Taketomi Y (2016). The roles of the secreted phospholipase A2 gene family in immunology. Adv Immunol.

[38] Wang Y, Song H, Miao Q, Wang Y, Qi J, Xu X, et al. PLA2G6 silencing suppresses melanoma progression and affects ferroptosis revealed by quantitative proteomics. Front Oncol. 2022;12. 10.3389/fonc.2022.819235 PMC8948425 35340268

[39] Oien DB, Pathoulas CL, Ray U, Thirusangu P, Kalogera E, Shridhar V. Repurposing quinacrine for treatment-refractory cancer. In: Seminars in Cancer Biology. Elsevier; 2021. p. 21–30. 10.1016/j.semcancer.2019.09.021 31562955

[40] Kumar M, Sarkar A (2022). Repurposing of anti-malarial drug quinacrine for cancer treatment: a review. Sci Pharm.

[41] Bryant J, Batis N, Franke AC, Clancey G, Hartley M, Ryan G (2019). Repurposed quinacrine synergizes with cisplatin, reducing the effective dose required for treatment of head and neck squamous cell carcinoma. Oncotarget.

[42] Friedman J, Nottingham L, Duggal P, Pernas FG, Yan B, Yang XP (2007). Deficient TP53 expression, function, and cisplatin sensitivity are restored by quinacrine in head and neck cancer. Clin Cancer Res.

[43] Li T, Wang W, Gong S, Sun H, Zhang H, Yang A-G (2018). Genome-wide analysis reveals TNFAIP8L2 as an immune checkpoint regulator of inflammation and metabolism. Mol Immunol.

[44] Sun H, Gong S, Carmody RJ, Hilliard A, Li L, Sun J (2008). TIPE2, a negative regulator of innate and adaptive immunity that maintains immune homeostasis. Cell.

[45] Lan G, Yu X, Sun X, Li W, Zhao Y, Lan J (2021). Comprehensive analysis of the expression and prognosis for TNFAIPs in head and neck cancer. Sci Rep.

[46] Bai K-H, Zhang Y-Y, Li X-P, Tian X-P, Pan M-M, Wang D-W (2022). Comprehensive analysis of tumor necrosis factor-α-inducible protein 8-like 2 (TIPE2): A potential novel pan-cancer immune checkpoint. Comput Struct Biotechnol J.

[47] Cao X, Zhang L, Shi Y, Sun Y, Dai S, Guo C (2013). Human tumor necrosis factor (TNF)-alpha-induced protein 8-like 2 suppresses hepatocellular carcinoma metastasis through inhibiting Rac1. Mol Cancer.

[48] Lorbek G, Lewinska M, Rozman D (2012). Cytochrome P450s in the synthesis of cholesterol and bile acids–from mouse models to human diseases. FEBS J.

[49] Kimbung S, Inasu M, Stålhammar T, Nodin B, Elebro K, Tryggvadottir H (2020). CYP27A1 expression is associated with risk of late lethal estrogen receptor-positive breast cancer in postmenopausal patients. Breast Cancer Res.

[50] Alfaqih MA, Nelson ER, Liu W, Safi R, Jasper JS, Macias E (2017). CYP27A1 loss dysregulates cholesterol homeostasis in prostate cancer CYP27A1 loss is involved in prostate cancer progression. Cancer Res.

[51] He S, Ma L, Baek AE, Vardanyan A, Vembar V, Chen JJ (2019). Host CYP27A1 expression is essential for ovarian cancer progression. Endocr Relat Cancer.

[52] Ma L, Wang L, Nelson AT, Han C, He S, Henn MA (2020). 27-Hydroxycholesterol acts on myeloid immune cells to induce T cell dysfunction, promoting breast cancer progression. Cancer Lett.

[53] Liang Z, Jiao W, Wang L, Chen Y, Li D, Zhang Z (2022). CYP27A1 inhibits proliferation and migration of clear cell renal cell carcinoma via activation of LXRs/ABCA1. Exp Cell Res.

[54] Pearce EL, Walsh MC, Cejas PJ, Harms GM, Shen H, Wang L-S (2009). Enhancing CD8 T-cell memory by modulating fatty acid metabolism. Nature.

[55] Ma X, Bi E, Lu Y, Su P, Huang C, Liu L (2019). Cholesterol induces CD8+ T cell exhaustion in the tumor microenvironment. Cell Metab.

[56] Yang W, Bai Y, Xiong Y, Zhang J, Chen S, Zheng X (2016). Potentiating the antitumour response of CD8+ T cells by modulating cholesterol metabolism. Nature.

[57] Hubler MJ, Kennedy AJ (2016). Role of lipids in the metabolism and activation of immune cells. J Nutr Biochem.

[58] Endo Y, Kanno T, Nakajima T (2022). Fatty acid metabolism in T-cell function and differentiation. Int Immunol.

[59] Ichise H, Tsukamoto S, Hirashima T, Konishi Y, Oki C, Tsukiji S (2022). Functional visualization of NK cell-mediated killing of metastatic single tumor cells. Elife.

[60] Niavarani SR, Lawson C, Bakos O, Boudaud M, Batenchuk C, Rouleau S (2019). Lipid accumulation impairs natural killer cell cytotoxicity and tumor control in the postoperative period. BMC Cancer.

[61] Yu W, Lei Q, Yang L, Qin G, Liu S, Wang D (2021). Contradictory roles of lipid metabolism in immune response within the tumor microenvironment. J Hematol OncolJ Hematol Oncol.

